# A flat petal as ancestral state for Ranunculaceae

**DOI:** 10.3389/fpls.2022.961906

**Published:** 2022-09-21

**Authors:** Pauline Delpeuch, Florian Jabbour, Catherine Damerval, Jürg Schönenberger, Susanne Pamperl, Maxime Rome, Sophie Nadot

**Affiliations:** ^1^Université Paris-Saclay, CNRS, AgroParisTech, Ecologie Systématique et Evolution, Orsay, France; ^2^Institut de Systématique, Evolution, Biodiversité (ISYEB), Muséum National d’Histoire Naturelle, CNRS, Sorbonne Université, EPHE, Université des Antilles, Paris, France; ^3^Université Paris-Saclay, INRAE, CNRS, AgroParisTech, Génétique Quantitative et Evolution-Le Moulon, Gif-sur-Yvette, France; ^4^Department of Botany and Biodiversity Research, University of Vienna, Vienna, Austria; ^5^Jardin du Lautaret, CNRS, Université Grenoble Alpes, Grenoble, France

**Keywords:** Ranunculaceae, ancestral state reconstruction, elaborate petals, floral morphology, petal development

## Abstract

Ranunculaceae comprise *ca.* 2,500 species (*ca.* 55 genera) that display a broad range of floral diversity, particularly at the level of the perianth. Petals, when present, are often referred to as “elaborate” because they have a complex morphology. In addition, the petals usually produce and store nectar, which gives them a crucial functional role in the interaction with pollinators. Its morphological diversity and species richness make this family a particularly suitable model group for studying the evolution of complex morphologies. Our aims are (1) to reconstruct the ancestral form of the petal and evolutionary stages at the scale of Ranunculaceae, (2) to test the hypothesis that there are morphogenetic regions on the petal that are common to all species and that interspecific morphological diversity may be due to differences in the relative proportions of these regions during development. We scored and analyzed traits (descriptors) that characterize in detail the complexity of mature petal morphology in 32 genera. Furthermore, we described petal development using high resolution X-Ray computed tomography (HRX-CT) in six species with contrasting petal forms (*Ficaria verna, Helleborus orientalis, Staphisagria picta, Aconitum napellus, Nigella damascena, Aquilegia vulgaris*). Ancestral state reconstruction was performed using a robust and dated phylogeny of the family, allowing us to produce new hypotheses for petal evolution in Ranunculaceae. Our results suggest a flat ancestral petal with a short claw for the entire family and for the ancestors of all tribes except Adonideae. The elaborate petals that are present in different lineages have evolved independently, and similar morphologies are the result of convergent evolution.

## Introduction

A key question in evolutionary biology is the origin of morphological diversity. The flower, the most striking innovation of angiosperms, is remarkable for its great morphological diversity despite a highly conserved ground plan: The floral receptacle bears, from outside in, sterile organs forming the perianth, male reproductive organs (stamens) forming the androecium, and female reproductive organs (carpels) forming the gynoecium. The perianth may be differentiated into a calyx formed by the sepals, and a corolla formed by the petals, or it may be undifferentiated. In this latter case, which most likely corresponds to the ancestral condition in angiosperms (Sauquet et al., 2017), the perianth organs are usually named tepals.

**FIGURE 1:**
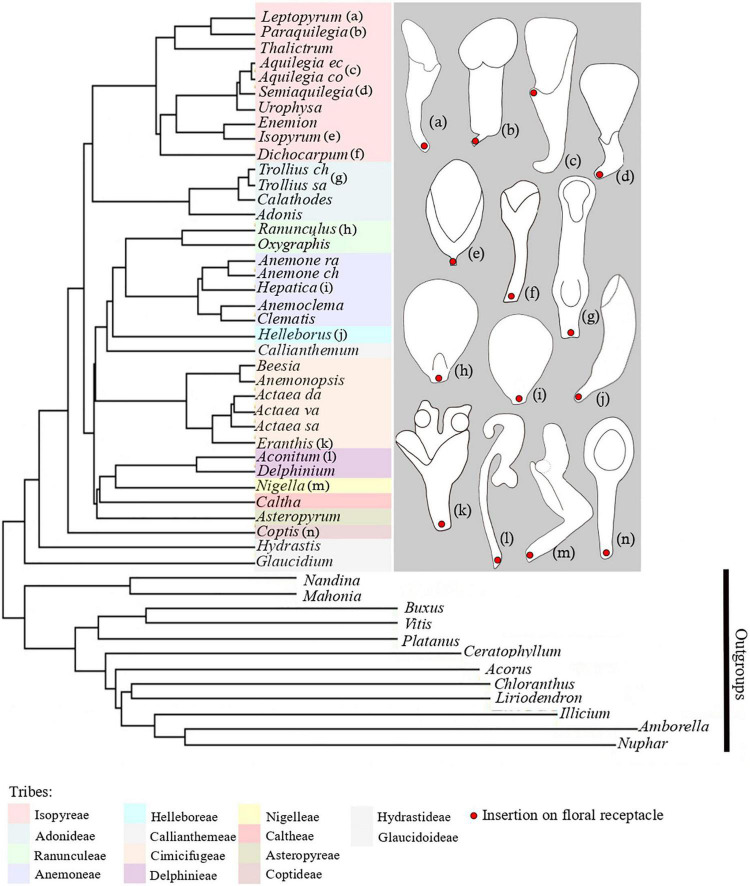
Phylogenetic tree of Ranunculaceae, including representatives of all tribes of the family. Phylogenetic tree and tribal classification are adapted from Zhai et al. (2019). Species names missing are: *Anemone raddeana, Anemone chinensis*, *Actaea dahurica, Actaea vaginata, Actaea asiatica, Trollius chinensis, Trollius saniculifolia, Aquilegia ecalcara, Aquilegia coerulea.* On the right, schematic line drawings of a selection of petals displaying the morphological diversity of petals in Ranunculaceae. Letters *a*-*n* refer to the position of the species in the phylogenetiv tree.

The order Ranunculales is among the most diverse angiosperm lineages with respect to floral organization (Carrive et al., 2020). The flowers display considerable variation in terms of number of floral organs, size, organ shape, and perianth differentiation. Both actinomorphic and zygomorphic flowers are recorded in this order (Sauquet et al., 2015). This variation makes this order an excellent model group for studies on the genetic basis of floral development and for the analysis of floral evolution in general, as suggested by Damerval and Becker (2017). The order consists of seven families. Its current taxonomic circumscription was already recognized in the first version of the APG classification (APG, 1998). It includes Ranunculaceae, Papaveraceae, Menispermaceae, Berberidaceae, Lardizabalaceae, Circaeasteraceae, and Eupteleaceae. The Ranunculaceae are the largest family with 2,525 species (Stevens, 2001, onward) and are well-known for the lack of morphological synapomorphies (Mangenot, 1973; Bonnier et al., 1990). The family is characterized by the presence, in many species, of so-called “elaborate” petals, i.e., petals with a complex, three-dimensional shape (Figure 1 and see Supplementary material 1 for illustrations of flowers). Moreover, these organs have the capacity to produce and store nectar, which gives them a major functional role in the interaction with pollinators and, thus, in the reproductive cycle of the plants. These nectar producing petals are traditionally called “Honigblätter” (Prantl, 1888), “Nektarblätter” (Janchen, 1949), or nectariferous petals (Leins and Erbar, 1997).

Several authors hypothesized that petals are of staminal origin in Ranunculaceae (Ronse De Craene and Smets, 1994, 1995; Erbar et al., 1999) mainly because stamen and petal primordia are indistinguishable at the earliest stages of development (e.g., Ren et al., 2011; Zhao et al., 2012b). Until recently, petals in Ranunculaceae were considered to have been repeatedly recruited from stamens during the evolutionary history of the family (Kosuge, 1994; Ronse De Craene, 2007). This view has recently been challenged by evo-devo studies of ABCE model gene expression in several Ranunculales species (reviewed in Damerval and Becker, 2017; Meaders et al., 2020) and by the Ranunculales-wide study on flower organization of Carrive et al. (2020). These studies revealed that the ancestral flower of Ranunculaceae likely had petals that were further lost repeatedly in several genera belonging to different tribes (*Anemone, Anemoclema, Enemion, Clematis, Caltha, Glaucidium, Hydrasti*s). They also suggested that the ancestral petal was already elaborate, allowing the storage of nectar. However, petal elaborations were considered in a single binary character, namely “Aspect of inner perianth organs” (petaloid and/or strongly modified). Because the three-dimensional form of petals is indeed quite complex in many genera of Ranunculaceae, refining the coding of petal elaborations was necessary to better capture their complexity. Petals display a great diversity across the family, but similar elaborations (such as spurs, bulges, or invaginations) may be observed in phylogenetically distant lineages although located on different parts of the petal (nearer to the proximal or to the distal part of the petal). For this reason, our working hypothesis is that petals could possibly be composed of several morphological regions each under the control of a different gene regulatory network, and each evolving independently.

Therefore, we designed three morphological descriptors to characterize the complexity of mature petal morphology across 32 genera (out of 55). In addition, we described and compared petal development using HRX-CT in six species with contrasting petal forms. Based on ancestral state reconstructions, we propose a new hypothesis for the ancestral petal form of Ranunculaceae and discuss the evolution of each three-dimensional characteristics in light of previous studies and of knowledge on the genetic bases of petal formation.

## Materials and methods

### Phylogenetic framework

Ancestral state reconstruction was performed using a plastome-based robust, well resolved, and dated genus-level phylogeny of the family (Zhai et al., 2019). Since our aim was not to challenge the phylogenetic hypothesis of Zhai et al. (2019), but to have a phylogenetic framework with branch lengths for ancestral state reconstruction, we used the same alignment and obtained a phylogram with the same topology using maximum likelihood, without testing branch support. The tree is presented in Supplementary material 2. It includes the five subfamilies and 14 tribes of Ranunculaceae (Isopyreae, Adonideae, Ranunculeae, Anemoneae, Helleboreae, Callianthemeae, Cimicifugeae, Delphinieae, Nigelleae, Caltheae, Asteropyreae, Coptideae, Hydrastideae, Glaucideae). Out of the 55 genera of Ranunculaceae, the 32 most speciose were sampled in this phylogeny.

### Literature survey

For each of the sampled genera we collected descriptions and photographs of the petals of as many species as possible, to record potential intrageneric morphological diversity. Intrageneric diversity in petal form occurs in the genera *Nigella, Eranthis, Aconitum*, and *Coptis.* To further explore the evolution of petal form at the genus-level, an in-depth study of petal form was carried out in *Coptis*, for which a recent genus-level phylogenetic framework was available (Xiang et al., 2016). Although the diversity in petal form, as well as the phylogenetic relationships among species, were described for *Eranthis* and *Nigella* in the literature, reliable phylogenetic frameworks could not be obtained for either genus, preventing us from reconstructing ancestral states.

Based on the work of Yao et al. (2019) in *Nigella*, there are four types of petal forms in this genus (Supplementary material 3A). The most common petal morphology is type 1 (represented here by the petal of *N. damascena*) in which the petal is clawed and has an invagination on the abaxial side in the median zone. Although the petals of *N. nigellastrum, N. integrifolia*, and *N. unguicularis*, which are different in form (devoid of invagination), belong to early diverging lineages within the genus (Yao et al., 2019), we chose *N. damascena* to represent the genus in the ancestral state reconstructions because this species has the most representative petal form of the genus. We cannot exclude though the possibility that this morphology is not ancestral in the genus.

According to the work of Huang and Zhang (2022) on *Eranthis*, the length of the claw varies among species and several species display pseudonectaries. There is always an outgrowth on the adaxial side. We chose the petal of *E. lobulata* to represent the genus in the ancestral state reconstructions as this species has the most representative petal form of the genus (Supplementary material 3B type 1).

We chose *Aconitum napellus* to represent the genus in the ancestral state reconstructions, because in the genus this species has been the most extensively studied regarding morphology, anatomy and development (Kosuge and Tamura, 1988; Erbar et al., 1999; Jabbour et al., 2009). In addition, this species was selected for our developmental study.

In the tribe Delphinieae, we characterized the morphology of dorsal petals only, as they are the rewarding petals. Additionally, we did not consider in our analyses the shape of the single petal of the flowers of *Delphinium* subg. *Consolida* (DuPasquier et al., 2021; Espinosa et al., 2021), resulting from the fusion of the two dorsalmost petal primordia at very early developmental stages (Jabbour and Renner, 2012). Petal morphology in this subgenus is derived within Delphinieae, and would have no effect on our ancestral state reconstructions.

### Plant material

We collected floral buds at different developmental stages for six species with contrasting petal forms: *Ficaria verna* Guettard, *Helleborus orientalis* L., *Staphisagria picta* J. Hill, *Aconitum napellus* L., *Nigella damascena* L., and *Aquilegia vulgaris* L. All species were collected in the Jardin Botanique de Launay (Orsay, France), Jardin Botanique du Lautaret (Haute-Alpes, France) and Wien botanical garden between July 2019 and August 2021 (Supplementary material 4). Buds were fixed in FAA (90% alcohol 70%, 5% formaldehyde, 5% acetic acid).

### Defining descriptors

**FIGURE 2:**
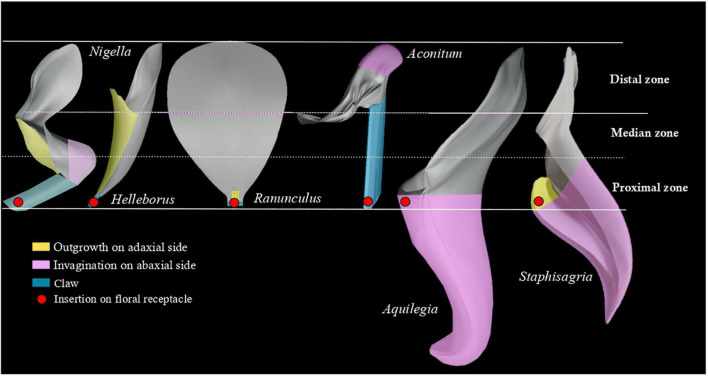
Three-dimensional models of six petals summarizing the morphological diversity of Ranunculaceae petals. The petals are oriented in space according to their insertion on the floral receptacle, and divided in three equal zones: proximal, median, and distal. The red dot indicates the insertion on the floral receptacle.

The petals were oriented in space according to the insertion on the floral receptacle and divided in three equal zones along the proximodistal axis: proximal, median, distal (Figure 2). The side of the petal facing the fertile organs is the adaxial side. The opposite side is the abaxial side.

We considered that petals are bilaterally symmetrical (or almost). Descriptors were therefore defined to account for morphological differentiation along the adaxial-abaxial and proximodistal axes only: “Outgrowth on adaxial side,” “Invagination on abaxial side” and “Clawed petal” (states: “absence,” “presence” and “non-applicable”). The latter descriptor refers to the narrowing of the petal toward the base. Non-applicable (NA) was used for flowers lacking petals. Each descriptor was coded for the whole petal and for each zone defined along the proximodistal axis.

In addition, we defined two functional characters focusing on traits related to nectar: “Presence of nectar in flowers,” “Location of nectar production,” and we added “Perianth differentiation” to check if the presence of nectar is associated with the presence of petals. Character states for the study species were obtained from the literature (including developmental data), floras, and personal observations. Data were also scored from online photographs if the identification could be trusted. All sources are listed in Supplementary material 5.

### Key nodes and ancestral state reconstructions

We selected the nodes (called “key nodes” from now on) in the phylogeny to reconstruct the hypothetical ancestral petal for the whole family and for the crown nodes of each of the 14 tribes.

Each character was analyzed separately. All characters defined (for the whole petal or for each zone) were discrete and had multiple states. We used the stochastic character mapping implemented in the function make.simmap of the PHYTOOLS R package (Revell et al., 2012), which allows for polymorphic/missing character states. make.simmap was run with 1,000 replicates per analysis under an all rates different model (“ARD”). We chose the model “ARD” based on the Akaike Information Criterion (Akaike, 1974).

### Comparing developmental sequences

To describe petal development, we used high resolution X-ray computed tomography (HRX-CT) on whole buds, and stereomicroscopy on dissected petals. For the HRX-CT analysis, floral buds were infiltrated with 1% phosphotungstic acid (PTA) and 70% ethanol to increase contrast, then critical point dried using an Em CPD300 (Leica Microsystems) and then scanned using a Zeiss MicroXCT-200 imaging system (Zeiss Microscopy) (see Staedler et al., 2013). Based on the raw scan data, 3D image-stacks were reconstructed using XMRECONSTRUCTOR v.8.1.6599 (Xradia) and on the resulting 3D models using AMIRA v.6.0 (Template Graphics Software Inc., San Diego, CA, USA). Virtual sections allowed us to visualize the form of floral organs during development, especially the form of elaborate petals. The development of stamens and carpels was used as a developmental reference to define key steps of floral development and compare in detail petal development among the six study species. In addition, we carried out dissections of FAA-fixed buds to extract the petals, which were observed using a stereomicroscope (Stemi 305 trino Zeiss) (Supplementary material 6).

## Results

### Mature petals

#### Classification of forms

On the abaxial side, we observed an invagination varying in width and depth among genera. Three main categories of invaginations were found: wide and shallow, like in *Coptis* (usually described as cups), wide and of medium depth in *Nigella* (described as urns), narrow and of medium depth in *Aconitum*, or narrow and deep like in *Aquilegia*. The latter are described as spurs in the literature (Supplementary material 7A). The terms that refer to width and depth (narrow, wide, deep and shallow) were used here as relative qualifiers (narrow relates to petal width, deep related to petal length), not as absolute values.

On the adaxial side, outgrowths of various lengths were recorded: short like in *Ranunculus* (described as nectary scales), long and not connected to the edges of the abaxial side like in *Nigella* (described as lips), long and connected to the edges of the abaxial side like in *Helleborus* (described as tubular or as funnels when the outgrowth is notched) (see Supplementary material 7B). Long relates here to petal length, it is not used as an absolute value.

A lip is an outgrowth that overlaps and closes a nectar chamber. A tubular or funnel petal is a single unit formed by the fusion of an adaxial outgrowth and an abaxial part of the same size.

#### Ancestral state reconstructions at the scale of *Coptis*

There are three types of petal forms in the genus *Coptis* (Supplementary material 8). Petals may be flat and linear, or flat and lanceolate embossed in the median zone, or spoon-shaped (i.e., with an enlarged distal zone, rounded in outline). The distal part has a wide and shallow invagination. Ancestral state reconstructions suggested a spoon-shaped petal as ancestral for the genus (Supplementary material 8). Petal shape in *Coptis* was coded based on the spoon-shaped petal of *C. trifolia*.

#### Ancestral state reconstructions at the scale of Ranunculaceae

**FIGURE 3:**
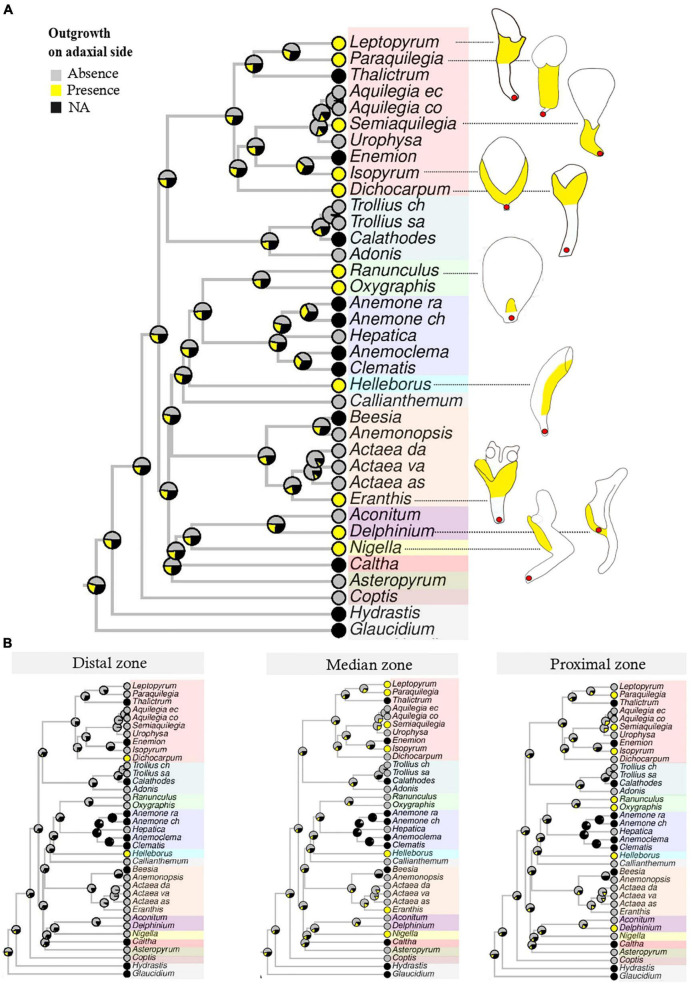
Bayesian ancestral state reconstructions for the character “Outgrowth on adaxial side.” Character states include “Absence,” “Presence,” “NA” (non-applicable, i.e., petals absent). **(A)** The character is coded for the petal as a whole, notwithstanding the zonation. Outgrowths are given in yellow in the line drawing. **(B)** The same character is coded for each zone (proximal, median, distal) separately.

When the three characters (“Outgrowth on adaxial side,” “Invagination on abaxial side” and “Claw”) were coded for the petal as a whole, notwithstanding petal zonation, the most probable form for the ancestral petal of Ranunculaceae was elaborate, clawed, without an outgrowth on the adaxial side (Figure 3A), but with an invagination on the abaxial side (Supplementary material 9, 10). When petals were divided in three zones along the proximodistal axis and each character was coded for each zone, ancestral state reconstructions suggested a flat and shortly clawed petal (without adaxial outgrowth and abaxial invagination) as ancestral for the family, as well as for the ancestors of all tribes except Adonideae (Figures 3B–D and Supplementary material 9B–D, 10B–D). For this latter tribe, the most likely ancestral form was a flat petal with a medium-sized claw (Supplementary material 11). The presence of a short claw in the proximal zone is therefore homologous for the whole family Ranunculaceae. All other elaborations, namely “Outgrowth on adaxial side” and “Invagination on abaxial side” are convergences among genera (Table 1) that are morphologically similar to some extent. For example, the spurred mature petals of *Aquilegia* (Isopyreae) and *Staphisagria* (Delphinieae) are similar in shape.

**TABLE 1:** Homology and convergence for the nine reconstructed ancestral states that take into account the location of morphological characters on petals.

Descriptors/Characters	Zones	Convergences	Homologies	Apomorphies
Outgrowth on adaxial side	Distal	Presence *(Dichocarpum, Helleborus)*		
	Median	Presence *(Leptopyrum, Paraquilegia, Semiaquilegia, Isopyrum, Helleborus, Eranthis, Nigella)*		
	Proximal	Presence *(Paraquilegia, Semiaquilegia, Helleborus)*	Presence (Ranunculeae tribe)	
Invagination on abaxial side	Distal	Presence *(Actaea, Coptis)*		
	Median	Presence *(Actaea, Nigella)*		
	Proximal	Presence *(Aquilegia, Urophysa, Delphinium)*		
Claw	Long			Presence *(Aconitum)*
	Median	Median *(Dichocarpum, Trolllius, Actaea, Asteropyrum, Coptis)*	Presence (Adonideae tribe)	
	Short		Presence (Ranunculaceae family)	

The localization of these elaborations varies among taxa. Striking examples are the tubular mature petals of *Helleborus* (Helleboreae), *Eranthis* (Cimicifugeae), *Leptopyrum*, and *Dichocarpum* (Isopyreae) that are very similar in form, but the outgrowth is located in the proximal (*Helleborus*), median (*Leptopyrum*), or distal zone (*Dichocarpum*).

### Comparing developmental sequences

**FIGURE 4:**
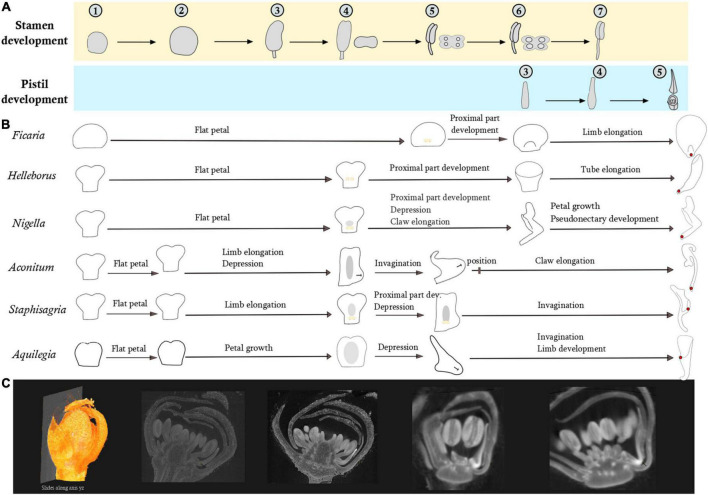
Comparison of petal development sequences of the six species representative of the morphological diversity using fertile organ development as reference. **(A)** Pistil and stamen development. Developmental stages of stamens: (1–2) Increase of the primordium size (2–3) filament/anther differentiation (3–4) differentiation of the two thecae (4–5) Connective differentiation (5–6) Stamen form is acquired, formation of the pollen sacs (6–7) Stamen elongation. Developmental stages of pistil: (3–4) Acquisition of the form (4–5) Ovules in the process of formation. **(B)** In yellow, development of bulges in the adaxial side. In gray, development of a depression in the abaxial side. **(C)** Example of a virtual section from a 3D floral bud reconstruction of *Staphisagria picta*.

The developmental sequences of the six study species are schematically presented in Figure 4. There is an arrest in petal development right after stamen inception. In all six species, the petals are flat at this stage but with a species-specific form. The petal blade in *H. orientalis, N. damascena, A. napellus*, and *S. picta* is bilobed in the distal zone, and clawed in the proximal zone. The primordium of *A. vulgaris* petal is bilobed, with an oval shape and no detectable narrowing. In *F. verna* the primordium is rounded. The delay observed in the resumption of petal development in the temporal frame established by the fertile organs development varies among species (Figure 4). In *H. orientalis, N. damascena*, and *F. verna*, the delay is longer than in the other genera investigated. In *H. orientalis* and *N. damascena*, petal development is resumed when the stamen thecae are differentiated (stage 4). In *F. verna*, petals resume their development when the connective of anthers is differentiated (stage 5). For *A. napellus*, *S. picta*, and *A. vulgaris*, the delay is shorter. Petals remain flat but start to differentiate during the interval between stage 2 and stage 3 of stamen differentiation.

In *N. damascena*, *A. napellus*, *S. picta*, and *A. vulgaris*, a depression appears abaxially in the median zone of the petal. As development proceeds, the depression deepens and turns into an invagination. This invagination remains shallow in *Nigella;* it becomes a little deeper in *A. napellus.* The final form of the invagination is acquired at stage 5 of stamen development. In the latest stages of development, there is an elongation of the claw and a homogeneous growth of the blade. In *S. picta* and *A. vulgaris*, invagination development proceeds until anthesis, forming a spur.

In *F. verna* and *N. damascena*, a small ridge develops on the adaxial side during connective differentiation, respectively at the base of the petal (*F. verna*) and just above the claw (*N. damascena*). We observed a differential growth between the adaxial side (growing faster) and the abaxial side. These outgrowths develop into a lip in *Nigella* and into a small nectary scale in *F. verna*. In *H. orientalis*, two bulges grow on the adaxial side and merge into a ridge. There is also a differential growth between the adaxial and abaxial sides, forming a short tube that further develops, resulting in a tubular mature petal.

### Nectar within flowers

**FIGURE 5:**
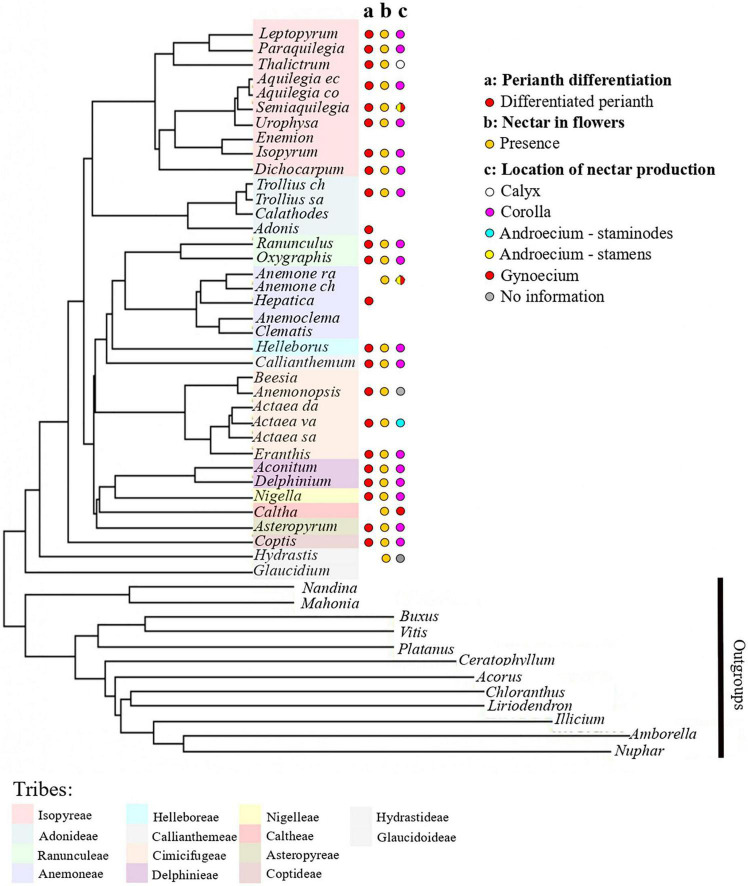
Phylogeny of the Ranunculaceae (adapted from Zhai et al., 2019) with the mapping of functional characters. (a) Perianth differentiation, (b) nectar in flowers, (c) location of nectar production. Color code for tribes as in Figure 3.

The results obtained concerning nectar location within flowers are presented in Figure 5. In Ranunculaceae flowers with petals, nectar tends to be produced and stored by the petals, whose elaborations allow nectar storage. Nectar production was also recorded in species belonging to genera with an undifferentiated perianth. It is the case in *Anemone nemorosa*, *Pulsatilla turczaninovii*, and *Caltha palustris*. Nectar production was recorded in *Clematis alpina*, which has a differentiated perianth contrary to most species of the genus *Clematis*, but in this species, nectar is produced by the fertile inner stamens or by the staminodes (Erbar and Leins, 2013).

## Discussion

### Mature petals

At the family scale, ancestral state reconstructions that do not take petal zonation into account suggest that the most likely ancestral petal is elaborate and clawed (Figure 3A and Supplementary material 9A, 10A). Conversely, when the petal is zoned and each character is coded for each zone, ancestral state reconstructions suggest a flat and shortly clawed ancestral petal for the entire family and for the ancestors of all tribes except Adonideae (Figures 3B–D and Supplementary material 9B–D, 10B–D, 11). The two types of reconstruction rely on two fundamentally different developmental hypotheses. In the first case, the whole petal is implicitly considered as a single developmental unit. In the second case, we assume that different developmental processes may take place in different sectors defined along the proximodistal axis. The fact that the two reconstructions give different ancestral state for the petal supports the existence of at least three morphogenetic regions in the developing petal. In addition, the ancestral state reconstructed from the zoned petal is more consistent with the situation observed in the rest of the order Ranunculales. Indeed, a previous study suggested a flat petal as the ancestral state for Ranunculales, for the sister family of Ranunculaceae, i.e., Berberidaceae, and for the closely related family Menispermaceae (Carrive et al., 2020). Furthermore, in the Berberidaceae, studies of petal development showed that the first stage of development is a flat petal (Su et al., 2021), similar to the result obtained in the present study for Ranunculaceae. Fine scale studies of gene regulatory networks at play during the development of the different morphological regions hypothesized here will be necessary to test this hypothesis in a representative selection of species.

All elaborations found in the family, namely the outgrowth on the adaxial side and the invagination on the abaxial side, should therefore be interpreted as convergences among genera. These convergences resulted in forms that are sometimes very similar. The convergent feature that evolved the most often was the outgrowth on the adaxial side, as shown in Figure 3. This outgrowth was present in all 10 genera (out of 22) with a differentiated perianth represented in the phylogeny and could possibly result from an adaptation to prevent nectar evaporation. Invaginations on the abaxial side in the form of a spur (deep and narrow) originated independently in the lineages leading to the genus *Aquilegia* and to the tribe Delphinieae (Supplementary material 9). Morphogenetic analyses have shown that the *Aquilegia* spur develops in two phases, proliferation, at the beginning of the development and anisotropic cell expansion afterward. The duration of this elongation phase is the major contributor to between species variability in spur length (Puzey et al., 2012). Comparative transcriptomic and genetic analyses have revealed genes and processes that could be involved in spur formation and curvature (Yant et al., 2015; Ballerini et al., 2019, 2020; Edwards et al., 2021). It would be interesting to obtain information on the cellular and genetic processes involved in spur morphogenesis in the tribe Delphinieae. This would shed light on the degree of convergence of this organ at several levels of phenotypic complexity.

### Nectaries or pseudonectaries?

Pseudonectaries, present in many species of angiosperms, are described as resembling nectar droplets, supposed to optically attract pollinators. In Ranunculaceae, such structures have been described in the genera *Eranthis*, *Trollius*, and *Nigella.* They are always domed, of contrasting pigmentation with the rest of the petal and present in the distal zone. Even if these structures mimic nectar that is not present on the bulge, nectar is present elsewhere on these petals, in the median or proximal zone. In this case, pseudonectaries not only attract pollinators but also help pollinators find the nectar that is sometimes hidden in a funnel-shaped outgrowth like in *Eranthis* (Huang and Zhang, 2022) or inside a nectar chamber like in *Nigella* (Weber, 1995). The expression of several genes involved in chloroplast development, cell division and wax formation have been found enriched in pseudonectary tissue in *N. damascena*. In particular, the inactivation of the *NdYAB5* gene results in the absence of pseudonectaries and have significant consequences on pollinator visitation (Liao et al., 2020). Interestingly in *Coptis*, in which the petals are described as nectariferous, except for *C. teeta* (Pandit and Babu, 1998), information on the precise location of nectar proved however difficult to obtain. For flat and lanceolate forms such as those observed in *C. aspleniifolia, C. laciniata*, and *C. occidentalis*, nectar is described as being located at the base of the petal (Li et al., 2013), while a study on *C. trifolia* mentions the presence of nectar within the shallow invagination located in the distal zone of the spoon-shaped petal (Li et al., 2013). This latter situation contrasts with the rest of *Coptis*, and even with the rest of Ranunculaceae, where nectar is described as produced in the proximal or median zone of the petal, but never in the distal zone. If the description provided for *C. trifolia* is indeed correct, our hypothesis is that there would have been a relocation of nectar production and storage within the petal in this species. An alternative hypothesis would be that the nectar is produced at the base of the petal, as in the other *Coptis* species for which nectar information is available (*C. aspleniifolia, C. laciniata*, and *C. occidentalis*), and that the distal zone of the petal would form a pseudonectary, exhibiting a cupped shape and contrasting color, as in the genera *Eranthis, Trollius*, and *Nigella*. If it is the case, this suggests that pseudonectaries could be either domed (like in *Eranthis, Trollius*, and *Nigella*) or cup-shaped (like in *Coptis*). In any case, it would be interesting to investigate these aspects by focusing on these genera.

### Nectar location

In Ranunculaceae with a differentiated perianth, nectar tends to be produced and stored by the petals, whose elaborations allow nectar storage. However, a survey of the literature shows that nectar production has been recorded in several species with petal-less flowers. The nectar-producing zones are then relocated in the fertile organs (carpels or stamens) or in staminodes. This is for example the case in the apetalous species *Anemone nemorosa* that produces nectar in the gynoecium. It is not very surprising since nectariferous tissues are known to be have various locations in plants (Fahn, 1979, 1988; Nepi, 2007) and pollinator-driven selection is very strong on these characters.

### Developmental sequences

All the species studied here have a flat petal at the beginning of development, in agreement with all studies already published on these species (Erbar et al., 1999; Tucker and Hodges, 2005; Zhao et al., 2011, 2012b; Yao et al., 2019). However, our study highlights the existence of slightly different petal shapes very early in development, even before the developmental stasis. The most common view according to which in the early stages of development the petal is flat, bilobed, and narrowed at the base, is not validated for all species. This is indeed the case for *H. orientalis, N. damascena, A. napellus*, and *S. picta*, but not for *A. vulgaris* and *F. verna* that do not display a narrowing at the base of the petals in the early developmental stages. This narrow region could have been considered as the sign of a future claw. However, while indeed no claw was observed in the mature petal of both *A. vulgaris* and *F. verna* without the basal narrowing, no claw could be observed in the Delphinieae species.

Using the development of fertile organs as a reference time point, we highlight a different pace in the developmental sequence among species, which is potentially indicative of heterochrony. This phenomenon concerns several steps of the development, from the resumption of growth to the differentiation of the peduncle or the initiation of the depression on the abaxial side. We observed that the depression becomes wider and turns into an invagination on the abaxial side of the petal in *A. napellus* and *N. damascena* and into a spur (deep and narrow invagination) in *S. picta* and *A. vulgaris*. A similar ancestral developmental mechanism could operate in the initial formation of the invagination in *Nigella* and the Delphinieae species, which are phylogenetically closely related. We observed the presence of ridges or bulges in the early stages of petal development in *F. verna*, *N. damascena*, and *H. orientalis*, which undergo differentiated growth in the different species, in the form of a nectary scale, a lip, or a tube, respectively (Leinfellner, 1958; Kosuge, 1994; Erbar et al., 1999). The ridge of *F. verna* and *N. damascena* is a continuous outgrowth at the base of the petal that develops faster than the blade. The bulges of *H. orientalis* are two small outgrowths, also located at the base of the petal, which then join, forming a ridge before growing faster than the blade. Therefore, outgrowths on the adaxial side that are described as nectary scales, can be obtained by the development of a ridge or bulges. This has been described in the tribe Ranunculeae (Zhao et al., 2012a). Indeed, the authors observed a ridge very similar to that of *Nigella* in the developmental sequences of *Ranunculus chinensis*, *Ceratocephala orthoceras, Halerpestes cymbalaria*, and *Oxygraphis glacialis*. In the present study, we observed a bulge at the base of the petal on the adaxial side in *Ficaria verna* (as in Erbar et al., 1999). For *Ranunculus sceleratus* and *Ranunculus bungei*, Zhao et al. (2012a) refer to a basal “crescent-shaped ridge” and “horseshoe-shaped ridge,” respectively.

This raises the question of the homology of this structure. The genus *Ranunculus* is particularly interesting because the petals of all species in the genus have a nectary scale that varies in form between species (Benson, 1940; Leinfellner, 1958). *Ranunculus* is the most speciose genus in Ranunculaceae, and is represented on all continents, so it could be a good model to address the question of developmental and environmental factors involved in the fine scale evolution of petal form. The form of the nectary scale could result from functional adaptation to pollinators, particularly to their mouthparts (Tian and Ren, 2019). In addition, this petal could be an excellent model to improve knowledge on the genes or gene networks involved in petal morphogenesis because the petal form of *Ranunculus* is relatively simple, the only elaboration being the nectary scale. Until now, studies on the gene networks underlying petal development in Ranunculaceae have been focused on *Aquilegia* (Yant et al., 2015; Ballerini et al., 2020) and *Nigella* (Gonçalves et al., 2013; Zhang et al., 2020; Deveaux et al., 2021). It would be interesting to investigate the expression patterns of such genes in a comparative developmental time frame as we have established in our study species.

## Conclusion

Our study suggests that the petal of Ranunculaceae is composed of different regions that may evolve independently from each other. Indeed, by decomposing the petal into zones along a proximodistal axis and an abaxial-adaxial axis, each coded separately, we propose that the ancestral petal of Ranunculaceae was flat and evolved repeatedly into various elaborate forms, contrary to previous hypotheses suggesting that the ancestral ranunculacean flower had elaborate petals. Elaborate petals would therefore be derived, with repeated convergences in forms.

Our comparisons of the floral on developmental sequences enlighten heterochrony as one possible cause of petal diversity. Moreover, it suggests that a similar ancestral developmental mechanism could operate in the initial formation of the invagination in *Nigella* and the Delphinieae species, which are phylogenetically closely related. It would be interesting to investigate whether the cellular and genetic mechanisms controlling the formation of the spur in *Aquilegia*, which is only distantly related with these species within Ranunculaceae, is similar or different. Our results on these species, representative of the diversity of petal forms in Ranunculaceae, pave the way for future studies focusing on gene regulatory networks involved in the morphogenesis of petal elaborations, including nectariferous tissues.

## Data availability statement

The original contributions presented in this study are included in the article/Supplementary material, further inquiries can be directed to the corresponding author/s.

## Author contributions

SN, FJ, CD, and PD designed the study. PD, SP, and JS performed the developmental analysis. PD performed the ancestral state reconstructions and wrote the first draft of the manuscript. MR helped with the plant material. All authors contributed to the article and approved the submitted version.
