# Supplementary material for: A flat petal as ancestral state for Ranunculaceae
Source: Front Plant Sci. 2022 Sep 21;13:961906. doi: 10.3389/fpls.2022.961906 (PMC9532948; doi:10.3389/fpls.2022.961906)
Supplement: Supplementary file 1 [file Data_Sheet_1.pdf]

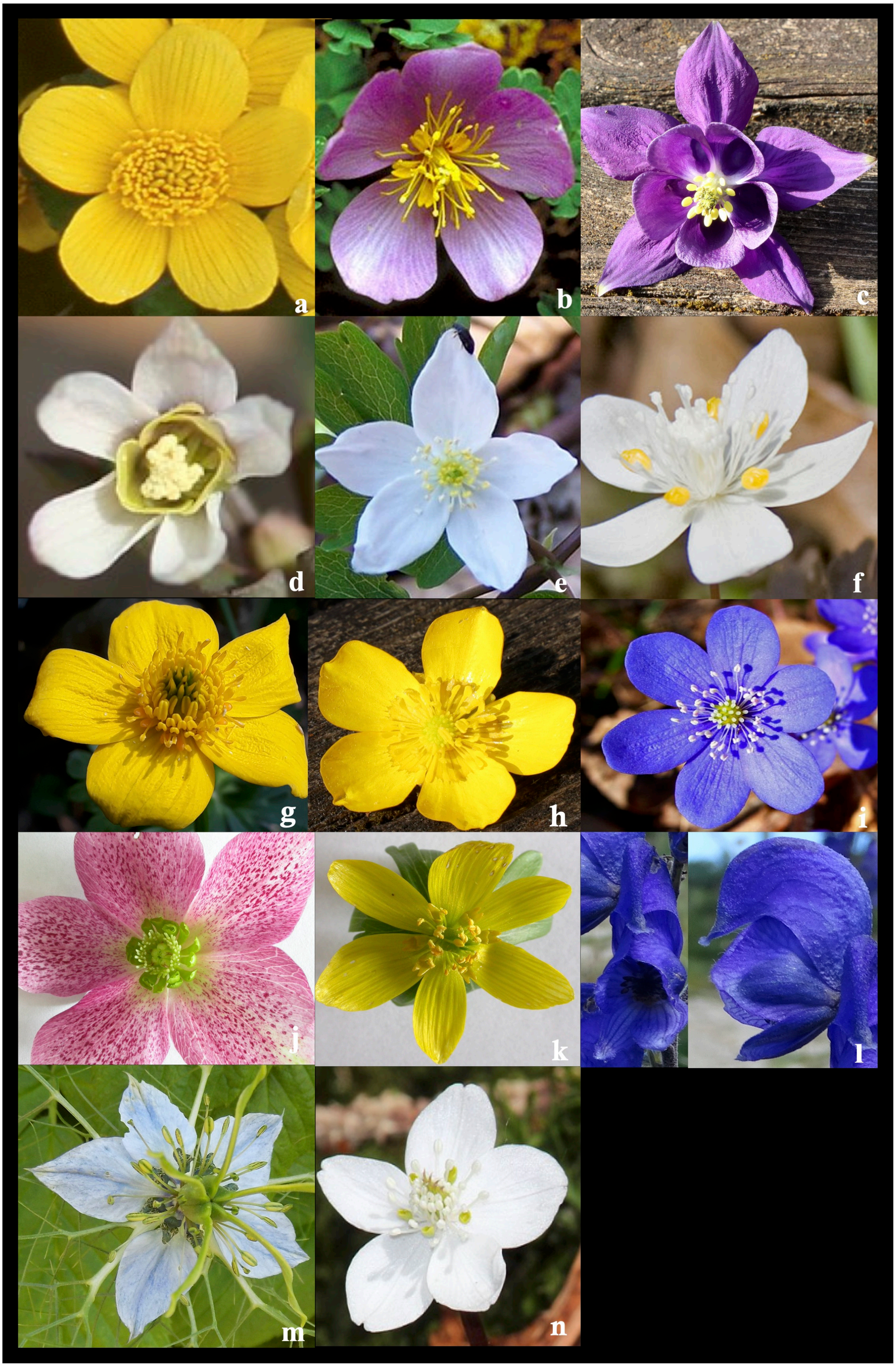

Supplementary material 1: Floral diversity in Ranunculaceae (the species are chosen following Figure 1): (a) *Leptopyrum fumarioides*, Wikimedia © Virginia; (b) *Paraquilegia microphylla*, Flora of China; (c) *Aquilegia vulgaris* © Antoine Plumerault; (d) *Semiaquilegia danxiashanensis*, Wikimedia © Alpsdake; (e) *Isopyrum thalictroides* Wikimedia © Opioła Jerzy; (f) *Coptis quinquefolia*, Wikimedia © Alpsdake; (g) *Trollius pumilus* © Rémi Petrolli; (h) *Ranunculus acris* © Pauline Delpeuch (i) *Hepatica nobilis*, Wikimedia © Bengt Nyman (j) *Helleborus orientalis* © Bruno Lascaux; (k) *Eranthis hyemalis* © Bruno Lascaux; (l) *Aconitum napellus* © Isabelle et Lucie Cavanié ; (m) *Nigella damascena* © Bruno Lascaux; (n) *Dichocarpum fargesii*, Wikimedia © ツルシロカネソウ
