# Supplementary material for: A flat petal as ancestral state for Ranunculaceae
Source: Front Plant Sci. 2022 Sep 21;13:961906. doi: 10.3389/fpls.2022.961906 (PMC9532948; doi:10.3389/fpls.2022.961906)
Supplement: Supplementary file 2 [file Data_Sheet_2.pdf]

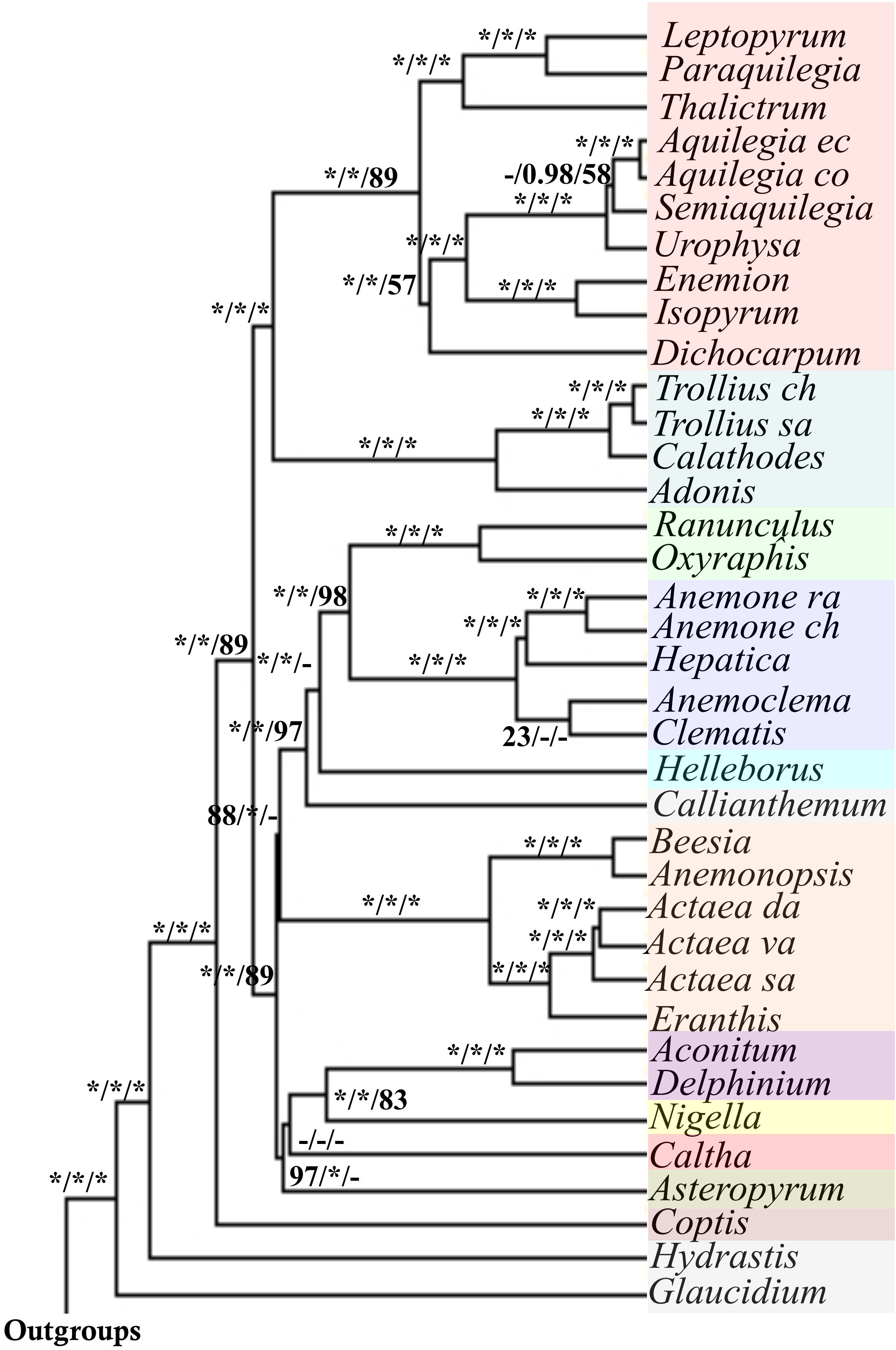

Supplementary material 2: Phylogenetic framework used in this study, obtained using the alignment of Zhai et al. (2019), based on plastomes, reconstructed using maximum likelihood. The topology is the same as in the original article of Zhai et al., support values from figure 2 of the original study are reported above nodes (from left to right: ML bootstrap values/Bayesian posterior probabilities/MP bootstrap value; asterisks indicate full support) . It includes the five subfamilies and 14 tribes of Ranunculaceae (Isopyreae, Adonideae, Ranunculeae, Anemoneae, Helleboreae, Callianthemeae, Cimicifugeae, Delphinieae, Nigelleae, Caltheae, Asteropyreae, Coptideae, Hydrastideae, Glaucideae).
