# Supplementary material for: A flat petal as ancestral state for Ranunculaceae
Source: Front Plant Sci. 2022 Sep 21;13:961906. doi: 10.3389/fpls.2022.961906 (PMC9532948; doi:10.3389/fpls.2022.961906)
Supplement: Supplementary file 3 [file Data_Sheet_3.pdf]

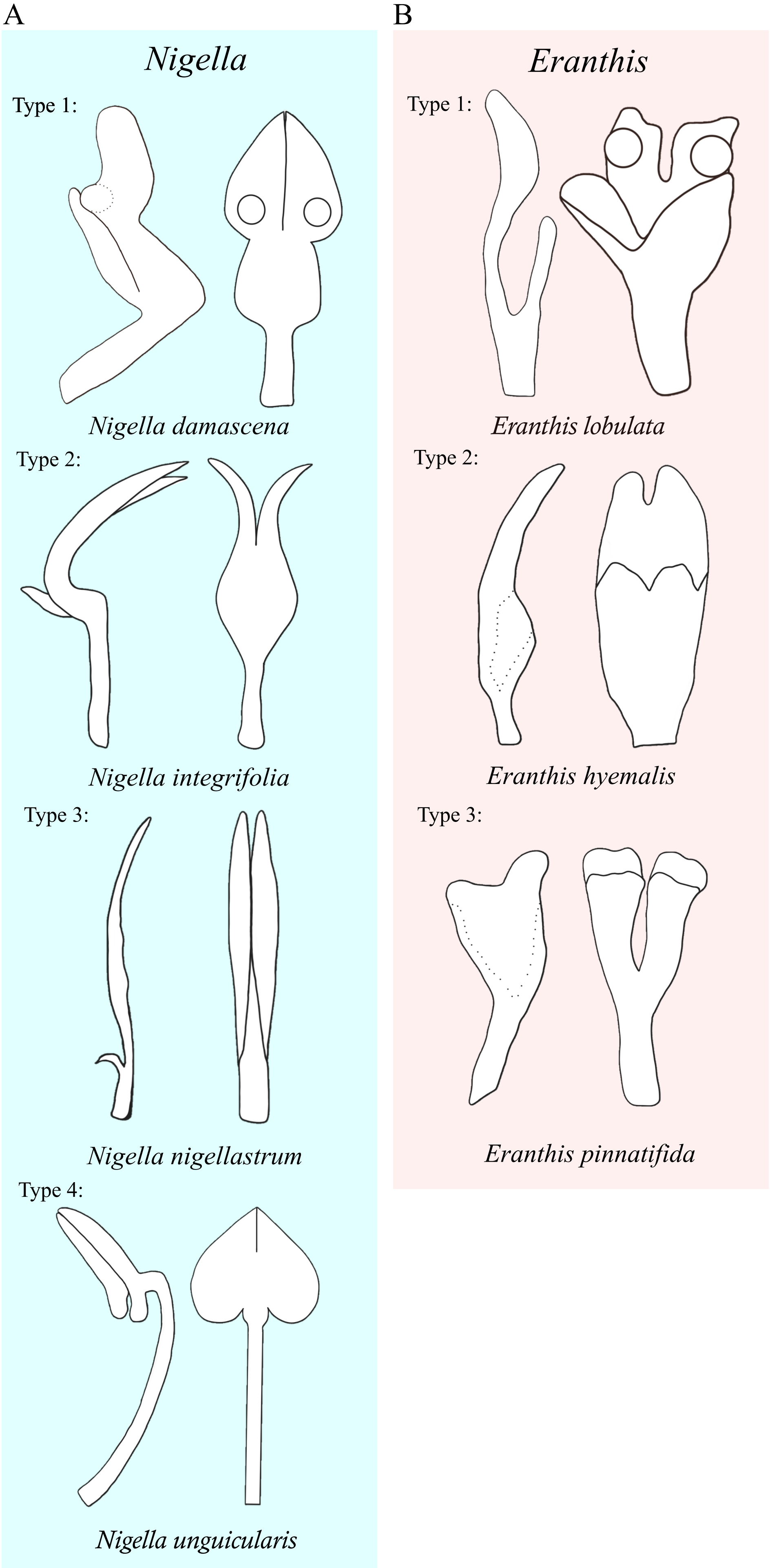

Supplementary material 3: Morphological variation of petals in *Nigella* (A) and *Eranthis* (B).
