# Supplementary material for: A flat petal as ancestral state for Ranunculaceae
Source: Front Plant Sci. 2022 Sep 21;13:961906. doi: 10.3389/fpls.2022.961906 (PMC9532948; doi:10.3389/fpls.2022.961906)
Supplement: Supplementary file 4 [file Data_Sheet_4.docx]

Supplementary material 4: Origin of plant material.

| **Species** | **Dates** | **Origin** |
| --- | --- | --- |
| *Aconitum napellus* | 07/2021 | Jardin Botanique du Lautaret (Haute-Alpes, France) |
| *Ficaria verna* | 03/2021 | Jardin Botanique de Launay (Orsay, France) |
| *Helleborus orientalis* | 12/2020 | Jardin Botanique de Launay (Orsay, France) PBL-005394 |
| *Staphisagria picta* | 09/2021 | Museum national d’Histoire naturelle (Paris, France) MNHN 16-57 |
| *Nigella damascena* | 08/2021 | Jardin Botanique de Launay (Orsay, France) JBL-00505b |
| *Aquilegia vulgaris* | 05/2019 | Botanical Garden of the University of Vienna (Austria) |
