# Supplementary material for: A flat petal as ancestral state for Ranunculaceae
Source: Front Plant Sci. 2022 Sep 21;13:961906. doi: 10.3389/fpls.2022.961906 (PMC9532948; doi:10.3389/fpls.2022.961906)
Supplement: Supplementary file 5 [file Data_Sheet_5.docx]

Supplementary material 5 – Sources used to score petal form and nectar location in selected species of Ranunculaceae (left column: genus; middle column: species and sources used for petal form; right colum: species and sources used for nectar location).

| **Genus** | **Petal form** | **Nectar location** |
| --- | --- | --- |
| *Anemone* | Absence  *A. chinensis, A. taipaiensis* (Zhai et al., 2019)  *A. blanda* (Missouri Botanical Garden)  *A. nemorosa* (Erbar and Leins, 2013; Zhang et al., 2013)  *A. montana, A. alpina* (Kubitzki et al., 2013; Yang et al., 2019)  *A. shikokiana* (Bian et al., 2018) | Absence  except for *A. nemorosa*  (Erbar and Leins, 2013; Antoń and Kamińska, 2015) |
| *Hepatica* | Absence  *H. henryi* (Zhai et al., 2019 ; Zhang et al., 2013)  Presence  *H. nobilis* (Carrive et al., 2020) | Absence  *H. nobilis* (Jagel, 2014) |
| *Clematis* | Absence  *C. macropetala* (Zhai et al., 2019)  *C. peterae* (REN et al., 2010; Zhang et al., 2013)  *C. apiifolia, C. vitalba, C. terniflora* (Carrive et al., 2020)  *C. stans, C. chrysocoma, C. recta* (Flora of China @ efloras.org)  *C. akebioides* (Yang et al., 2019)  *C. socialis, C. heracleifolia, C. integrifolia* (Zhang et al., 2013)  Presence  *C. macropetala* (Yang et al., 2019)  *C. alpina* (Zhang et al. 2013) | Absence  *C. macropetala*, *C. peterae,* *C. apiifolia, C. vitalba, C. terniflora* (Carrive et al. 2020)  Presence  *C. socialis* (Wall et al., 2003), *C. akebioides* (Yang et al., 2019)  *C. stans* (Dohzono and Suzuki, 2002) |
| *Anemoclema* | Absence (Zhai et al., 2019) | - |
| *Ranunculus* | Presence (Benson, 1940) | Presence (Benson, 1940) |
| *Oxygraphis* | Presence (Zhai et al., 2019) | Presence (Flora of China @ efloras.org) |
| *Helleborus* | Presence  *H. thibetanus* (Zhai et al., 2019)  *H. foetidus, H. niger* (Carrive et al., 2020)  *H. viridis* (Kurth et al. 2012)  *H. orientalis* (personal observations) | Presence (Flora of China @ efloras.org)  (Kurth) |
| *Callianthemum* | Presence  *C. taipaicum* (Zhai et al., 2019)  *C. anemonoides* (Carrive et al. 2020)  *C. coriandrifolium* (personal observations) | Presence (Carrive et al. 2020; personal observations) |
| *Actaea* | Absence  *A. simplex* (Huang and Zhang, 2022 ; Pellmyr, 1986)  *A. cordifolia* Absence (Flora of North America @ efloras.org), Presence (Pellmyr, 1986b)  Presence  *A. dahurica, A. vaginata, A. asiatica* (Zhai et al., 2019)  *A. racemosa* (Yang et al., 2019)  *A. purpurea* (Chang et al., 2022)  *A. rubra* (Lehmann and Sattler, 1994) *A. europaea* (Hiepko,1965; Lehmann and Sattler, 1994)  *A. arizona* (Pellmyr, 1985b, Lehmann and Sattler, 1994)  *A. japonica* (Kosuge and Tamura, 1988; Lehmann and Sattler, 1994)  Discussions on the differentiation of the perianth because sometimes difficult to distinguish it from staminodes | Absence (Pellmyr, 1986a) (Kubitzki et al., 2013; Carrive et al., 2020) |
| *Eranthis* | Presence (Huang and Zhang, 2022)  *E. stellata* (Zhai et al., 2019)  *E. hyemalis, E. cilicica* (Carrive et al. 2020) | Presence (Huang and Zhang, 2022) |
| *Beesia* | Absence  *B. calthifolia* (Zhai et al., 2019) | - |
| *Anemonopsis* | *A.macrophylla* (Plantes et botanique : le site dédié au monde végétal, 2020) |  |
| *Nigella* | Presence (Yao et al., 2019) | Presence (Yao et al., 2019) |
| *Delphinium* | Presence  *D. ajacis* (Zhai et al. 2019)  *D. cheilantum, D. peregrinum* (Carrive et al., 2020)  *D. elatum* (Antoń and Kamińska, 2015) | Presence (Flora of China @ efloras.org |
| *Aconitum* | Presence  *A. kusnezoffii* (Zhai et al. 2019; Flora of China)  *A. napellus, A. lycoctonum* (personal observations) | Presence  (Flora of China @ efloras.org); personal observations) |
| *Caltha* | Absence  *C. palustris* (Zhai et al. 2019; personal observations)  *C. leptosepala* (http://worldfloraonline.org/ ) | Presence (Zhai et al. 2019) |
| *Asteropyrum* | Presence  *A. cavaleriei* (Zhai et al., 2019; Carrive et al., 2020) | Presence (Carrive et al. 2020) |
| *Trollius* | Presence  *T. chinensis* (Zhai et al. 2020, Carrive et al. 2020)  *T. saniculifolia* (Tucker and Hodges, 2005)  *T. europaeus, T. asiaticus, T. macropetalus, T. acaulis, T. japonicus, T. ledebourii, T. ranunculoides, T. pumilus, T. farreri, T. lilacinus, T. yunnanensis, T. altaicus, T. buddae* (Flora of China @ efloras.org)  *T. riederianus* (Flora of North America @ efloras.org) | Presence (Flora of China @ efloras.org)  *Trollius buddae* (Liao et al. 2020)  *T. europaeus* (Pellmyr et al. 1989) |
| *Calathodes* | Absence  *C. oxycarpa* (Zhai et al. 2020)  *C. palmata* (Baillon 1871, Tucker and Hodges, 2005) | No information |
| *Adonis* | Presence  *A. sutchuenensis* (Zhai et al. 2020, <http://worldfloraonline.org/>)  *A. vernalis* (Zhang et al. 2013) | Absence  *A. vernalis* (Denisow et al. 2014) |
| *Aquilegia* | Presence  *A. coerulea* (Zhai et al. 2020), *A.*  *ecalcarata* (Zhai et al. 2020, Carrive et al. 2020, Wang and Chen, 2007), *A. atrata, A. chrysantha* (Carrive et al. 2020, Tucker and Hodges, 2005, Flora of China @ efloras.org), *A. vulgaris* (Anton and Kaminska, 2014)*, A. buergeriana* (Kosuge et Tamura 1989, Tucker and Hodges, 2005), A. oxysepala (Wang and Chen, 2007) | Presence  (Tucker and Hodges, 2005, Carrive et al. 2020)  Absence  *A. ecarlata* (Tucker and Hodges, 2005, Hodges and Arnold, 1995; Fior et al., 2013) |
| *Semiaquilegia* | Presence  *S. adoxoides* (Tucker and Hodges, 2005) | Presence  *S. adoxoides* (Tucker and Hodges, 2005) |
| *Urophysa* | Presence  *U. rockii, U.* *henryi* (Zhai et al. 2020; Wang and Chen, 2007) | No information |
| *Isopyrum* | Presence  *I. manshuricum* (Zhang et al. 2013; Zhai et al. 2020), *I. thalictroides* (Zhang et al. 2013; Carrive et al. 2020) | Presence  *I. thalictroides* (Carrive et al. 2020) |
| *Enemion* | Absence  *E. raddeanum* (Zhang et al. 2013; Zhai et al. 2020), *E. occidentale* (Tucker and Hodegs, 2005) | No information |
| *Dichocarpum* | Presence  *D. fargesii* (Zhai et al. 2019, Flora of China @ efloras.org), *D. stoloniferum* (Carrive et al. 2020), *D. dalzielii, D. sutchuenense* (Wang and Chen, 2007), *D. hagiangense, D. trifoliolatum, D. pterigionocaudatum, D. auriculatum, D. nipponicum* (Nguyen et al., 2020) | Presence  *D. fargesii, D. stoloniferum* (Ren et al. 2011 ; Carrive et al. 2020) |
| *Paraquilegia* | Presence  *P. anemonoides* (Zhai et al., 2019 ; Carrive et al. 2020), *P. microphylla* (Wang and Chen, 2007) | Presence  *P. anemonoides* (Zhai et al., 2019 ; Carrive et al. 2020) |
| *Leptopyrum* | Presence  *L. fumarioides* (Wang and Chen, 2007, Zhang et al. 2013, Zhai et al. 2019) | No information |
| *Thalictrum* | Absence  *T. minus* (Zhai et al. 2019, Carrive et al. 2020), *T. aquilegiifolium, T. javanicum* (Carrive et al. 2020), *T. petaloideum, T.* *robustum* (Wang and Chen, 2007; Zhang et al., 2013) | Absence  *T. minus*, *T. aquilegiifolium, T. javanicum* (Kaplan and Mulcahy, 1971 ; Carrive et al., 2020) |
| *Coptis* | Presence  *C. chinensis, C. quinquefolia, C. japonica, C. teeta, C. lutescens, C. omeinsis, C. deltoidae, C. quinquesecta, C. laciniata, C. occidentalis, C. aspleniifolia, C. ramosa, C. trifoliolata, C. morii, C. groenlandica, C. trifolia* (Xiang et al., 2016; Zhai et al. 2019; Carrive et al. 2020) | Presence  *C. laniata, C. occidentalis, C. aspleniifolia, C. trifolia* (Li et al., 2013)  *C. chinensis* (Flora of China @ efloras.org), *C. quinquefolia* (Carrive et al. 2020) |
| *Hydrastis* | Absence  *H. canadensis* (Zhai et al. 2019; Carrive et al. 2020) | Presence  *H. canadensis* (Liu et al., 2019) |
| *Glaucidium* | Absence  *G. palmatum* (Zhai et al. 2019; Carrive et al. 2020) | No information |

Literature cited:

Antoń, S., and Kamińska, M. (2015). Comparative floral spur anatomy and nectar secretion in four representatives of Ranunculaceae. *Protoplasma* 252, 1587–1601. doi: 10.1007/s00709-015-0794-5.

Benson, L. (1940). The North American Subdivisions of *Ranunculus*. *Am. J. Bot.* 27, 799–807. doi: 10.2307/2436909.

Bian, F. H., Chen, P., and Yu, W. Y. (2018). Breeding system of *Anemone shikokiana* and the influential factors of fruit-setting ratio in different habitats. *Plant Biosyst. - Int. J. Deal. Asp. Plant Biol.* 152, 445–452. doi: 10.1080/11263504.2017.1297336.

Carrive, L., Domenech, B., Sauquet, H., Jabbour, F., Damerval, C., and Nadot, S. (2020). Insights into the ancestral flowers of Ranunculales. *Bot. J. Linn. Soc.* 194, 23–46. doi: 10.1093/botlinnean/boaa031.

Chang, X.-P., Zhang, J.-D., Li, X.-F., Huang, L., Tian, X.-H., Ren, Y., et al. (2022). Morphological divergence and the Quaternary speciation of *Actaea purpurea* (Ranunculaceae) and its relatives. *J. Syst. Evol.* 60, 43–54. doi: 10.1111/jse.12667.

Dohzono, I., and Suzuki, K. (2002). Bumblebee-pollination and temporal change of the calyx tube length in *Clematis stans* (Ranunculaceae). *J. Plant Res.* 115, 355–359. doi: 10.1007/s10265-002-0046-6.

Erbar, C., and Leins, P. (2013). Nectar production in the pollen flower of *Anemone nemorosa* in comparison with other Ranunculaceae and *Magnolia* (Magnoliaceae). *Org. Divers. Evol.* 13, 287–300. doi: 10.1007/s13127-013-0131-9.

Flora of China @ efloras.org Available at: http://www.efloras.org/flora_page.aspx?flora_id=2 [Accessed May 5, 2022].

Huang, Z., and Zhang, X. (2022). Floral nectaries and pseudonectaries in *Eranthis* (Ranunculaceae): petal development, micromorphology, structure and ultrastructure. *Protoplasma*. doi: 10.1007/s00709-022-01738-1.

Jagel, A. (2014). *Hepatica nobilis* – Leberblümchen (Ranunculaceae), Blume des Jahres 2013. 6.

Kosuge, K., and Tamura, M. (1988). Morphology of the petal in *Aconitum*. *Bot. Mag. Tokyo* 101, 223–237. doi: 10.1007/BF02488601.

Kubitzki, K., Rohwer, J. G., and Bittrich, V. (2013). *Flowering Plants · Dicotyledons: Magnoliid, Hamamelid and Caryophyllid Families*. Springer Science & Business Media.

Kurth, C. Occurrence and diversity of yeasts in floral nectar of *Helleborus viridis* L. 39.

Lehmann, N. L., and Sattler, R. (1994). Floral development and homeosis in *Actaea rubra* (Ranunculaceae). *Int. J. Plant Sci.* 155, 658–671. doi: 10.1086/297205.

Liu, H., Ma, J., and Li, H. (2019). Transcriptomic and microstructural analyses in *Liriodendron tulipifera* Linn. reveal candidate genes involved in nectary development and nectar secretion. *BMC Plant Biol.* 19, 531. doi: 10.1186/s12870-019-2140-0.

Missouri Botanical Garden Available at: https://www.missouribotanicalgarden.org/ [Accessed May 29, 2022].

Nguyen, M. T., Trinh, N. B., Tran, T. T. V., Thanh, T. D., Phan, L. K., and Pham, V. T. (2020). *Dichocarpum hagiangense*—a new species and an updated checklist of Ranunculaceae in Vietnam. *PeerJ* 8, e9874. doi: 10.7717/peerj.9874.

Pellmyr, O. (1986a). The pollination ecology of two nectarless *Cimicifuga* sp. (Ranunculaceae) in North America. *Nord. J. Bot.* 6, 713–723. doi: 10.1111/j.1756-1051.1986.tb00472.x.

Pellmyr, O. (1986b). Three pollination morphs in *Cimicifuga* *simplex*; incipient speciation due to inferiority in competition. *Oecologia* 68, 304–307. doi: 10.1007/BF00384804.

Plantes et botanique : le site dédié au monde végétal (2020). *Plantes Bot.* Available at: https://www.plantes-botanique.org/ [Accessed May 29, 2022].

Ren, Y., Chang, H.-L., and Endress, P. K. (2010). Floral development in Anemoneae (Ranunculaceae). *Bot. J. Linn. Soc.* 162, 77–100. doi: 10.1111/j.1095-8339.2009.01017.x.

Tucker, S. C., and Hodges, S. A. (2005). Floral Ontogeny of Aquilegia, Semiaquilegia, and Enemion (Ranunculaceae). *Int. J. Plant Sci.* 166, 557–574. doi: 10.1086/429848.

Wall, M. A., Timmerman-Erskine, M., and Boyd, R. S. (2003). Conservation impact of climatic variability on pollination of the federally endangered plant, *Clematis socialis* (Ranunculaceae). *Southeast. Nat.* 2, 11–24. doi: 10.1656/1528-7092(2003)002[0011:CIOCVO]2.0.CO;2.

Wang, W., and Chen, Z.-D. (2007). Generic level phylogeny of Thalictroideae (Ranunculaceae) — implications for the taxonomic status of *Paropyrum* and petal evolution. *Taxon* 56, 811–821. doi: 10.2307/25065863.

Yang, M., Deng, G.-C., Gong, Y.-B., and Huang, S.-Q. (2019). Nectar yeasts enhance the interaction between *Clematis akebioides* and its bumblebee pollinator. *Plant Biol.* 21, 732–737. doi: 10.1111/plb.12957.

Yao, X., Zhang, W., Duan, X., Yuan, Y., Zhang, R., Shan, H., et al. (2019). The making of elaborate petals in *Nigella* through developmental repatterning. *New Phytol.* 223, 385–396. doi: 10.1111/nph.15799.

Zhai, W., Duan, X., Zhang, R., Guo, C., Li, L., Xu, G., et al. (2019). Chloroplast genomic data provide new and robust insights into the phylogeny and evolution of the Ranunculaceae. *Mol. Phylogenet. Evol.* 135, 12–21. doi: 10.1016/j.ympev.2019.02.024.

Zhang, R., Guo, C., Zhang, W., Wang, P., Li, L., Duan, X., et al. (2013). Disruption of the petal identity gene APETALA3-3 is highly correlated with loss of petals within the buttercup family (Ranunculaceae). *Proc. Natl. Acad. Sci.* 110, 5074–5079. doi: 10.1073/pnas.1219690110.
