# Supplementary material for: A flat petal as ancestral state for Ranunculaceae
Source: Front Plant Sci. 2022 Sep 21;13:961906. doi: 10.3389/fpls.2022.961906 (PMC9532948; doi:10.3389/fpls.2022.961906)
Supplement: Supplementary file 6 [file Data_Sheet_6.pdf]

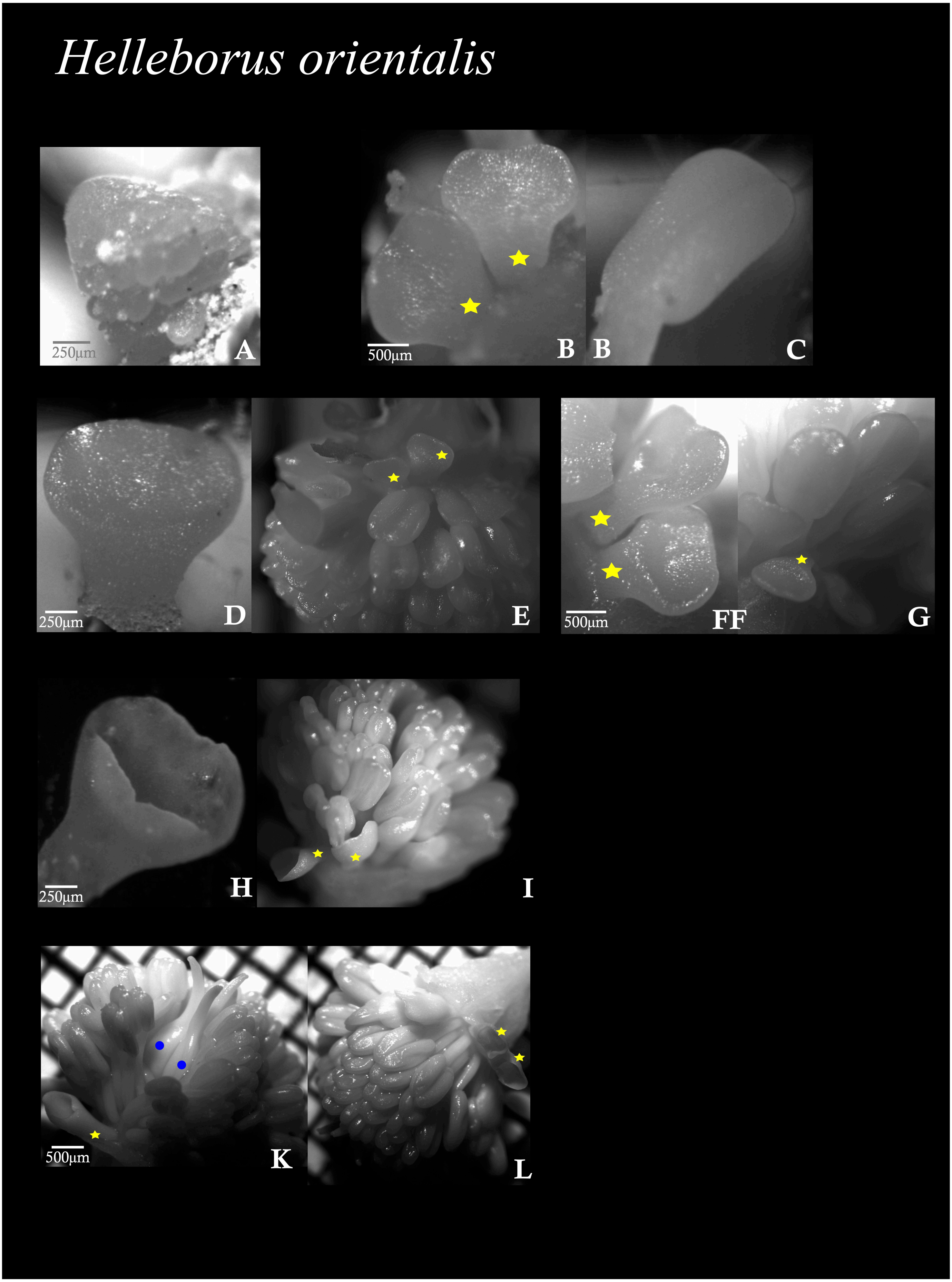

Supplementary material 6: Pictures of dissected petals under stereomicroscopy. Yellow stars indicate the rewarding petals, blue dots indicate the pistls.
