# Supplementary material for: A flat petal as ancestral state for Ranunculaceae
Source: Front Plant Sci. 2022 Sep 21;13:961906. doi: 10.3389/fpls.2022.961906 (PMC9532948; doi:10.3389/fpls.2022.961906)
Supplement: Supplementary file 7 [file Data_Sheet_7.pdf]

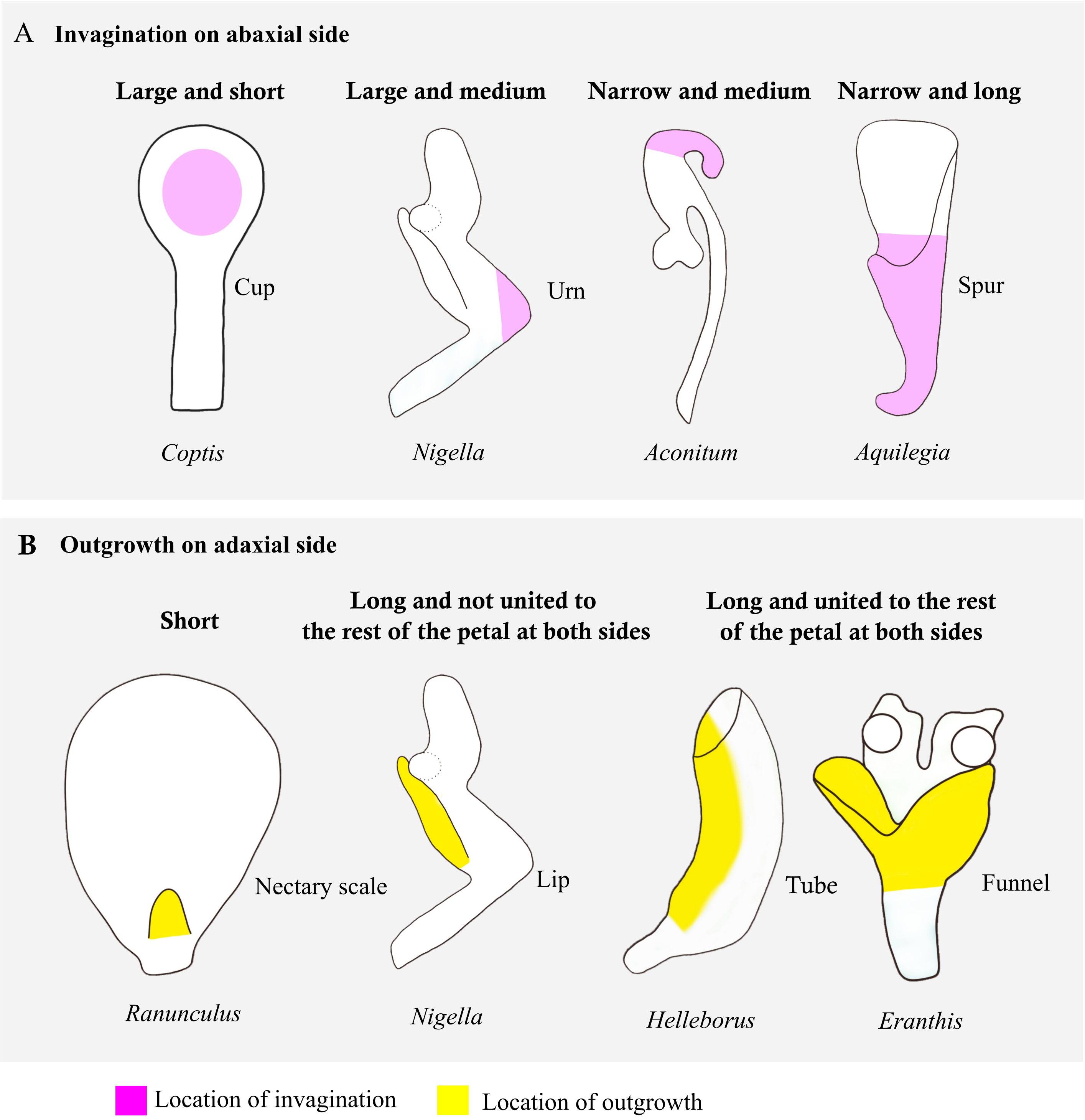

Supplementary material 7: Terminology of the different types of invaginations and outgrowths (A and B) used in the literature. The coloured parts in pink and yellow indicate the location of petal invaginations (A) and petal outgrowths (B), respectively.
In A: "Large" and "narrow" refer to the diameter of the invagination. "Narrow" refers to a diameter of 25% or less of the petal size, "large" is more than 25%. The terms "short", "medium", and "long" refer to its depth. Relative proportions define them. An invagination smaller than the opening diameter is considered as "short", equal or up to twice as long is considered "medium", and more than twice is considered "long".
In B: "Short" and "long" refer to the size of the outgrowth. "Short" corresponds to 25% of the petal and "long" corresponds to more than 25%.
