# Supplementary material for: A flat petal as ancestral state for Ranunculaceae
Source: Front Plant Sci. 2022 Sep 21;13:961906. doi: 10.3389/fpls.2022.961906 (PMC9532948; doi:10.3389/fpls.2022.961906)
Supplement: Supplementary file 8 [file Data_Sheet_8.pdf]

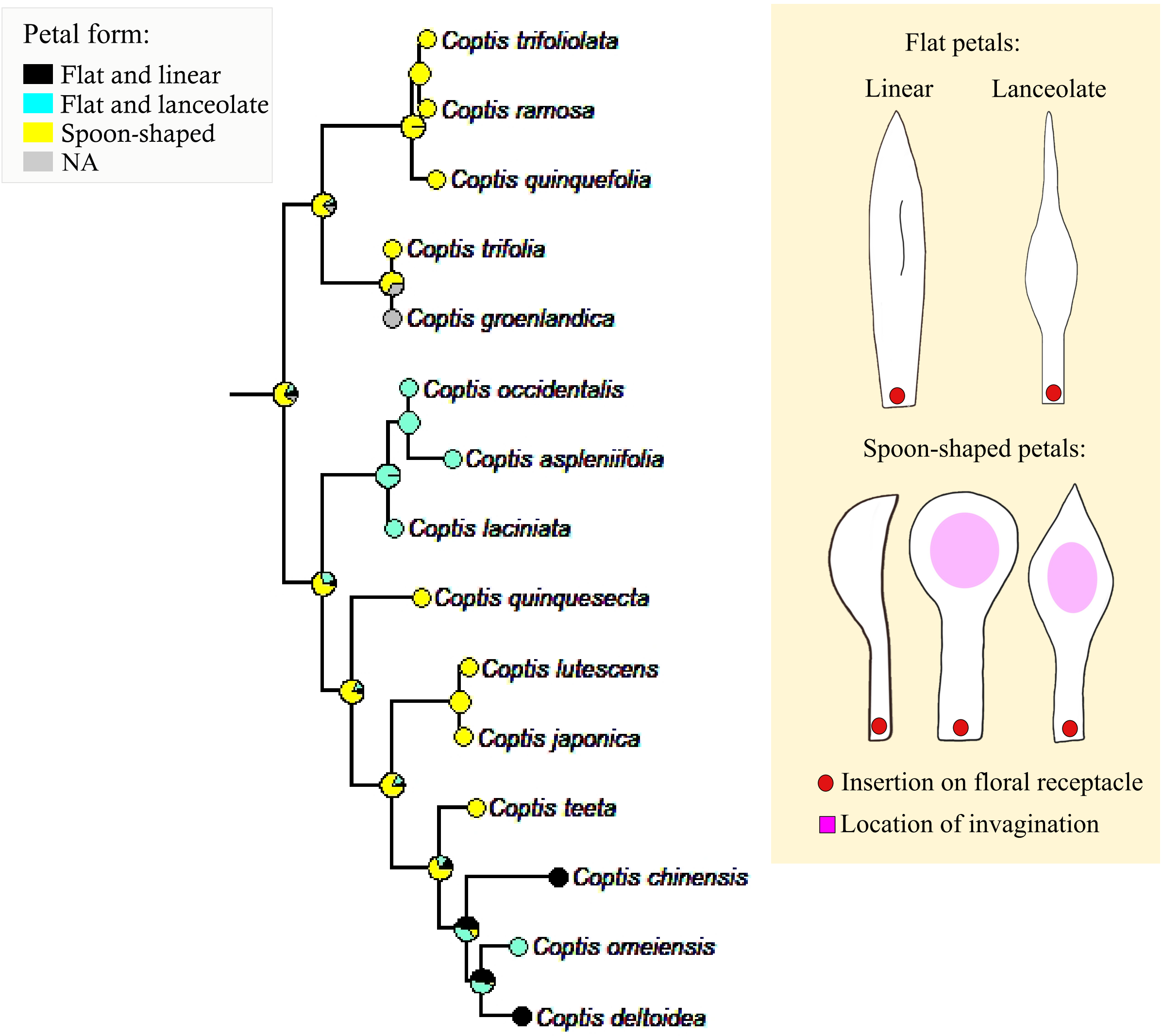

Supplementary material 8: Genus *Coptis* - Bayesian ancestral state reconstructions for the character “petal form”. Ancestral states are “Linear”, “Lanceolate”, “Spoon”, “NA” (non applicable, i.e., no information on *C. groenlandica*). Phylogenetic tree and morphological data used are adapted from Xiang et al. 2016. On the right, simplified shape of a selection of petals displaying a samle of the morphological diversity in Coptis. Red dots indicate the insertnio point of petals on the floral receptacle. The pink zone is the location of the invagination.
