# Supplementary material for: A flat petal as ancestral state for Ranunculaceae
Source: Front Plant Sci. 2022 Sep 21;13:961906. doi: 10.3389/fpls.2022.961906 (PMC9532948; doi:10.3389/fpls.2022.961906)
Supplement: Supplementary file 9 [file Data_Sheet_9.pdf]

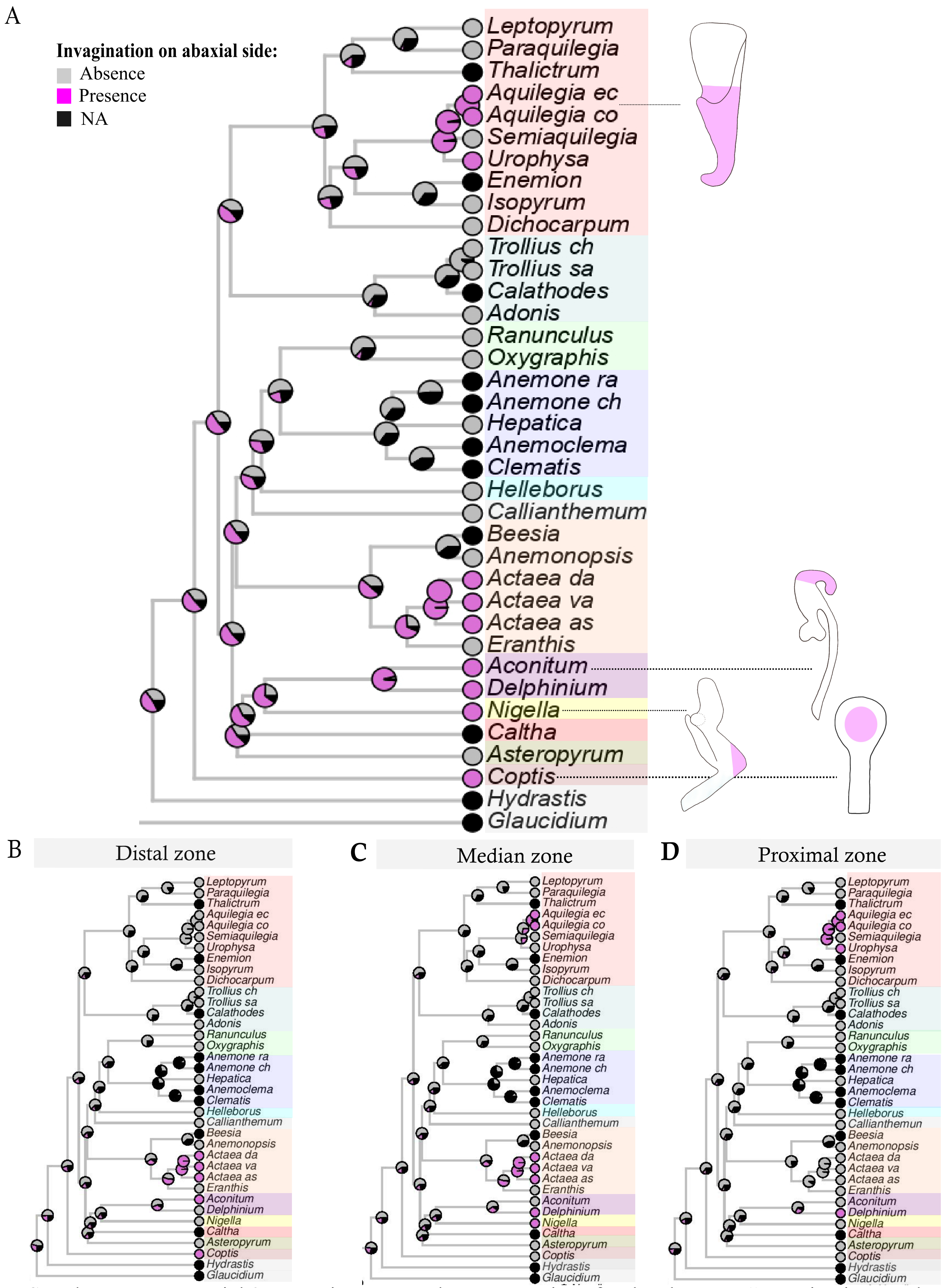

Supplementary material 9: Bayesian ancestral reconstructions for the character "Invagination on abaxial side". Ancestral states are "Absence", "Presence", "NA" (non applicable, i.e., petals absent)
A: The character is coded for the petal as a whole, notwithstanding the zonation. B:
B. The same character is coded for each zone (proximal, median, distal). Colour code for tribes as in figure 1.
