# Supplementary material for: A flat petal as ancestral state for Ranunculaceae
Source: Front Plant Sci. 2022 Sep 21;13:961906. doi: 10.3389/fpls.2022.961906 (PMC9532948; doi:10.3389/fpls.2022.961906)
Supplement: Supplementary file 10 [file Data_Sheet_10.pdf]

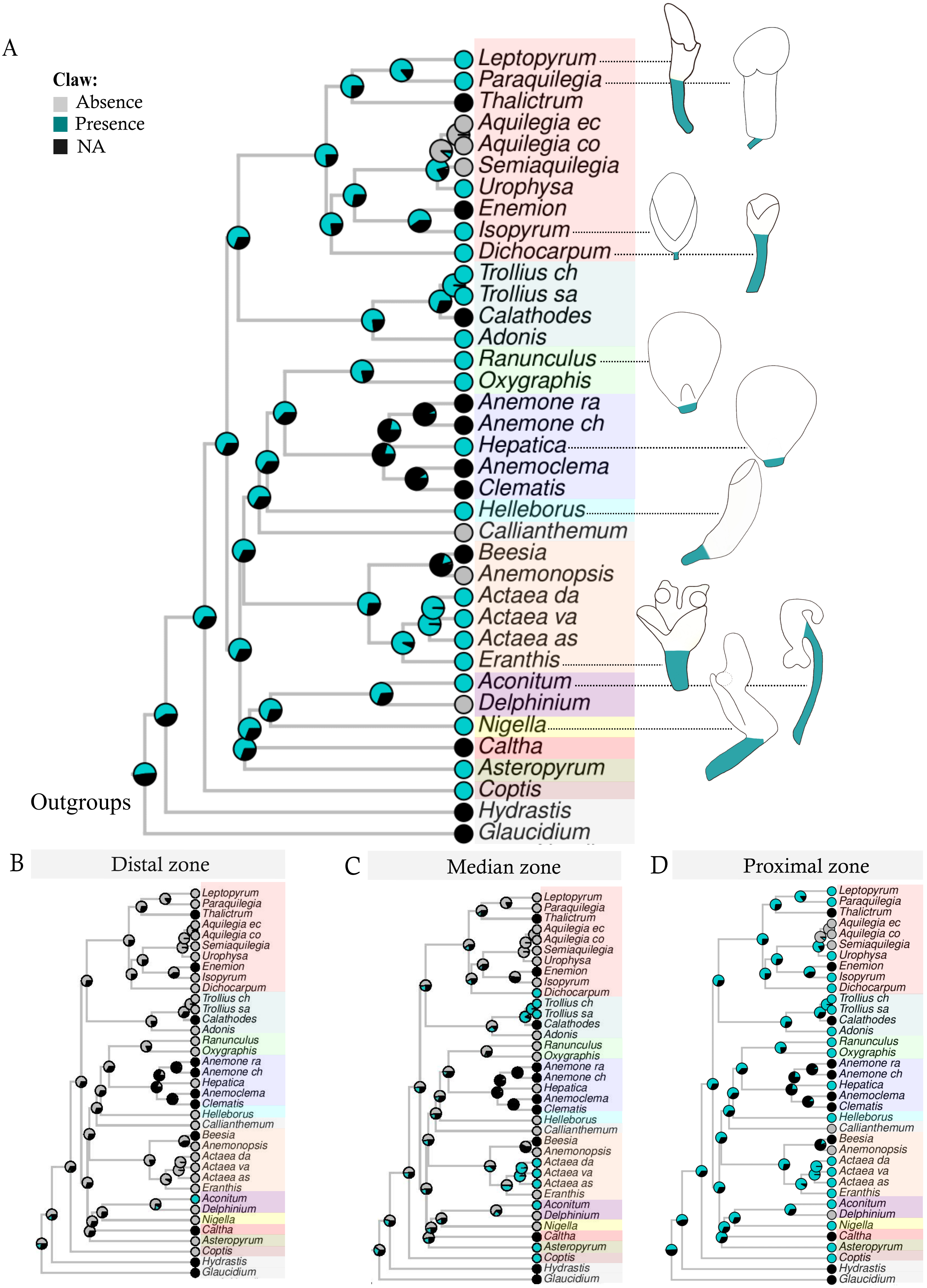

Supplementary material 10: Ancestral state reconstructions for the character "Claw" using Bayesian model. Ancestral states are "Absence", "Presence", "NA" (non applicable, i.e., petals absent). A: The character is coded for the petal as a whole, notwithstanding the zonation. B: The same character is coded for each zone (proximal, median, distal). The colour code for tribes is the same as in figure 1.

Node labels and numerical results of central state reconstructions

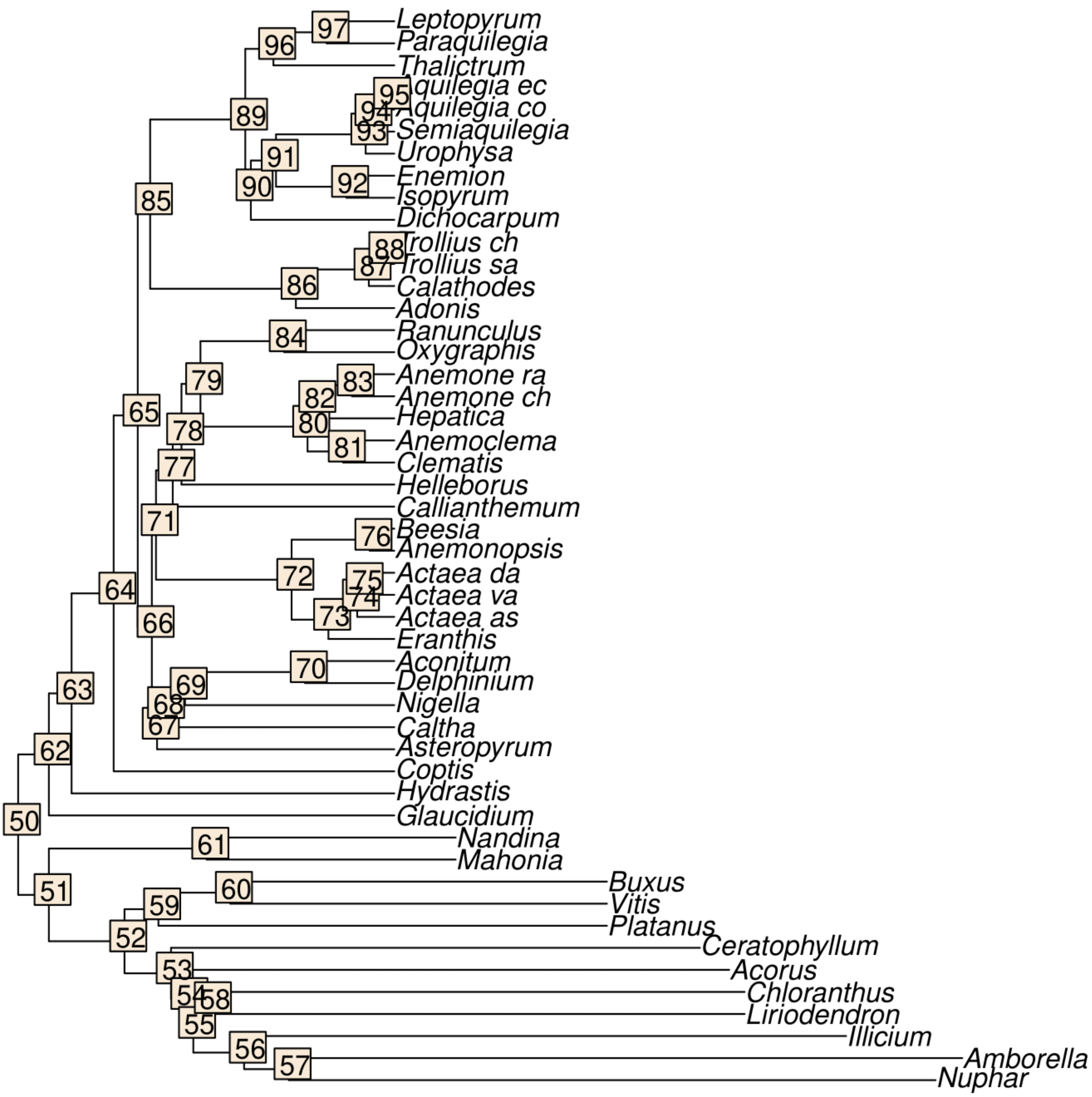

Invagination on abaxial side

Distal zone

	0	1	2
50	0.329	0.329	0.342
51	0.479	0.243	0.278
52	0.601	0.143	0.256
53	0.605	0.114	0.281
54	0.627	0.109	0.264
55	0.618	0.103	0.279
56	0.634	0.104	0.262
57	0.625	0.120	0.255
58	0.628	0.102	0.270
59	0.613	0.136	0.251
60	0.633	0.129	0.238
61	0.654	0.116	0.230
62	0.457	0.249	0.294
63	0.551	0.196	0.253
64	0.613	0.144	0.243
65	0.636	0.138	0.226
66	0.645	0.133	0.222
67	0.646	0.139	0.215
68	0.643	0.151	0.206
69	0.616	0.193	0.191
70	0.595	0.294	0.111
71	0.636	0.136	0.228
72	0.587	0.300	0.113
73	0.465	0.514	0.021
74	0.024	0.976	0.000
75	0.013	0.987	0.000
76	0.608	0.014	0.378
77	0.650	0.107	0.243
78	0.640	0.098	0.262
79	0.617	0.083	0.300
80	0.263	0.004	0.733
81	0.135	0.001	0.864
82	0.261	0.003	0.736
83	0.092	0.001	0.907
84	0.723	0.071	0.206
85	0.640	0.130	0.230
86	0.684	0.065	0.251
87	0.638	0.002	0.360
88	0.962	0.000	0.038
89	0.678	0.065	0.257
90	0.680	0.066	0.254
91	0.699	0.075	0.226
92	0.586	0.015	0.399
93	0.748	0.247	0.005
94	0.707	0.291	0.002
95	0.002	0.998	0.000
96	0.660	0.059	0.281
97	0.817	0.048	0.135

Median zone

	0	1	2
50	0.329	0.329	0.342
51	0.479	0.243	0.278
52	0.601	0.143	0.256
53	0.605	0.114	0.281
54	0.627	0.109	0.264
55	0.618	0.103	0.279
56	0.634	0.104	0.262
57	0.625	0.120	0.255
58	0.628	0.102	0.270
59	0.613	0.136	0.251
60	0.633	0.129	0.238
61	0.654	0.116	0.230
62	0.457	0.249	0.294
63	0.551	0.196	0.253
64	0.613	0.144	0.243
65	0.636	0.138	0.226
66	0.645	0.133	0.222
67	0.646	0.139	0.215
68	0.643	0.151	0.206
69	0.616	0.193	0.191
70	0.595	0.294	0.111
71	0.636	0.136	0.228
72	0.587	0.300	0.113
73	0.465	0.514	0.021
74	0.024	0.976	0.000
75	0.013	0.987	0.000
76	0.608	0.014	0.378
77	0.650	0.107	0.243
78	0.640	0.098	0.262
79	0.617	0.083	0.300
80	0.263	0.004	0.733
81	0.135	0.001	0.864
82	0.261	0.003	0.736
83	0.092	0.001	0.907
84	0.723	0.071	0.206
85	0.640	0.130	0.230
86	0.684	0.065	0.251
87	0.638	0.002	0.360
88	0.962	0.000	0.038
89	0.678	0.065	0.257
90	0.680	0.066	0.254
91	0.699	0.075	0.226
92	0.586	0.015	0.399
93	0.748	0.247	0.005
94	0.707	0.291	0.002
95	0.002	0.998	0.000
96	0.660	0.059	0.281
97	0.817	0.048	0.135

Proximal zone

	0	1	2
50	0.330	0.310	0.360
51	0.521	0.178	0.301
52	0.680	0.075	0.245
53	0.717	0.066	0.217
54	0.715	0.068	0.217
55	0.715	0.062	0.223
56	0.725	0.043	0.232
57	0.696	0.047	0.257
58	0.707	0.066	0.227
59	0.716	0.047	0.237
60	0.717	0.047	0.236
61	0.677	0.052	0.271
62	0.497	0.206	0.297
63	0.566	0.138	0.296
64	0.644	0.093	0.263
65	0.659	0.079	0.262
66	0.695	0.066	0.239
67	0.705	0.068	0.227
68	0.700	0.072	0.228
69	0.713	0.082	0.205
70	0.674	0.232	0.094
71	0.693	0.060	0.247
72	0.771	0.030	0.199
73	0.886	0.017	0.097
74	0.970	0.009	0.021
75	0.972	0.009	0.019
76	0.593	0.002	0.405
77	0.692	0.047	0.261
78	0.679	0.048	0.273
79	0.656	0.046	0.298
80	0.243	0.002	0.755
81	0.106	0.000	0.894
82	0.243	0.001	0.756
83	0.086	0.000	0.914
84	0.724	0.047	0.229
85	0.683	0.072	0.245
86	0.746	0.034	0.220
87	0.661	0.003	0.336
88	0.978	0.001	0.021
89	0.711	0.106	0.183
90	0.710	0.110	0.180
91	0.658	0.181	0.161
92	0.653	0.025	0.322
93	0.050	0.949	0.001
94	0.050	0.949	0.001
95	0.000	1.000	0.000
96	0.727	0.060	0.213
97	0.838	0.037	0.125

State of character:

0 = absence (white)

1 = presence (pink)

2= no petals (black)

Outgrowth on adaxial side

Distal zone

	0	1	2
50	0.363	0.336	0.301
51	0.509	0.231	0.260
52	0.659	0.105	0.236
53	0.669	0.075	0.256
54	0.675	0.066	0.259
55	0.674	0.071	0.255
56	0.719	0.064	0.217
57	0.709	0.060	0.231
58	0.687	0.065	0.248
59	0.686	0.088	0.226
60	0.683	0.074	0.243
61	0.712	0.053	0.235
62	0.481	0.243	0.276
63	0.535	0.194	0.271
64	0.626	0.120	0.254
65	0.647	0.109	0.244
66	0.660	0.103	0.237
67	0.673	0.091	0.236
68	0.679	0.083	0.238
69	0.708	0.076	0.216
70	0.826	0.027	0.147
71	0.664	0.104	0.232
72	0.799	0.030	0.171
73	0.917	0.008	0.075
74	0.978	0.002	0.020
75	0.978	0.002	0.020
76	0.577	0.006	0.417
77	0.666	0.104	0.230
78	0.648	0.107	0.245
79	0.639	0.081	0.280
80	0.189	0.003	0.808
81	0.076	0.003	0.921
82	0.199	0.003	0.798
83	0.051	0.002	0.947
84	0.774	0.034	0.192
85	0.662	0.101	0.237
86	0.728	0.030	0.242
87	0.647	0.006	0.347
88	0.973	0.002	0.025
89	0.697	0.115	0.188
90	0.699	0.124	0.177
91	0.724	0.076	0.200
92	0.630	0.018	0.352
93	0.982	0.003	0.015
94	0.991	0.002	0.007
95	1.000	0.000	0.000
96	0.693	0.075	0.232
97	0.837	0.032	0.131

Median zone

	0	1	2
50	0.339	0.308	0.353
51	0.535	0.168	0.297
52	0.601	0.150	0.249
53	0.613	0.156	0.231
54	0.602	0.165	0.233
55	0.622	0.145	0.233
56	0.590	0.146	0.264
57	0.593	0.161	0.246
58	0.617	0.137	0.246
59	0.627	0.131	0.242
60	0.613	0.152	0.235
61	0.640	0.132	0.228
62	0.517	0.166	0.317
63	0.548	0.149	0.303
64	0.582	0.140	0.278
65	0.604	0.137	0.259
66	0.577	0.166	0.257
67	0.586	0.159	0.255
68	0.584	0.152	0.264
69	0.603	0.171	0.226
70	0.655	0.182	0.163
71	0.581	0.162	0.257
72	0.657	0.165	0.178
73	0.712	0.197	0.091
74	0.846	0.130	0.024
75	0.864	0.115	0.021
76	0.525	0.050	0.425
77	0.592	0.136	0.272
78	0.582	0.140	0.278
79	0.577	0.122	0.301
80	0.186	0.025	0.789
81	0.077	0.008	0.915
82	0.191	0.027	0.782
83	0.065	0.002	0.933
84	0.664	0.153	0.183
85	0.608	0.144	0.248
86	0.646	0.145	0.209
87	0.610	0.058	0.332
88	0.930	0.031	0.039
89	0.636	0.135	0.229
90	0.645	0.145	0.210
91	0.631	0.181	0.188
92	0.571	0.121	0.308
93	0.754	0.240	0.006
94	0.735	0.262	0.003
95	0.968	0.032	0.000
96	0.604	0.152	0.244
97	0.691	0.223	0.086

Proximal zone

	0	1	2
50	0.341	0.320	0.339
51	0.553	0.164	0.283
52	0.601	0.135	0.264
53	0.602	0.151	0.247
54	0.592	0.148	0.260
55	0.602	0.149	0.249
56	0.641	0.129	0.230
57	0.624	0.145	0.231
58	0.606	0.149	0.245
59	0.600	0.138	0.262
60	0.605	0.150	0.245
61	0.629	0.134	0.237
62	0.533	0.175	0.292
63	0.575	0.152	0.273
64	0.605	0.145	0.250
65	0.627	0.120	0.253
66	0.627	0.138	0.235
67	0.614	0.153	0.233
68	0.626	0.141	0.233
69	0.613	0.156	0.231
70	0.709	0.165	0.126
71	0.618	0.144	0.238
72	0.648	0.178	0.174
73	0.739	0.196	0.065
74	0.833	0.152	0.015
75	0.851	0.133	0.016
76	0.516	0.054	0.430
77	0.605	0.149	0.246
78	0.585	0.159	0.256
79	0.575	0.140	0.285
80	0.177	0.025	0.798
81	0.077	0.007	0.916
82	0.177	0.026	0.797
83	0.054	0.008	0.938
84	0.691	0.170	0.139
85	0.623	0.121	0.256
86	0.625	0.150	0.225
87	0.595	0.054	0.351
88	0.945	0.026	0.029
89	0.624	0.155	0.221
90	0.623	0.172	0.205
91	0.644	0.168	0.188
92	0.589	0.123	0.288
93	0.771	0.223	0.006
94	0.758	0.237	0.005
95	0.988	0.012	0.000
96	0.602	0.146	0.252
97	0.698	0.185	0.117

State of character:

0 = absence (white)

1 = presence (yellow)

2= no petals (black)

Claw

	Distal zone			Median zone			Proximal zone		
	0	1	2	0	1	2	0	1	2
50	0.333	0.326	0.341	0.314	0.342	0.344	0.069	0.407	0.524
51	0.525	0.185	0.290	0.407	0.264	0.329	0.631	0.179	0.190
52	0.714	0.058	0.228	0.535	0.203	0.262	0.930	0.032	0.038
53	0.723	0.038	0.239	0.542	0.193	0.265	0.954	0.023	0.023
54	0.721	0.034	0.245	0.550	0.180	0.270	0.956	0.023	0.021
55	0.730	0.031	0.239	0.555	0.177	0.268	0.954	0.025	0.021
56	0.737	0.033	0.230	0.608	0.144	0.248	0.960	0.023	0.017
57	0.736	0.029	0.235	0.598	0.147	0.255	0.967	0.021	0.012
58	0.729	0.028	0.243	0.573	0.171	0.256	0.947	0.026	0.027
59	0.752	0.042	0.206	0.580	0.171	0.249	0.946	0.025	0.029
60	0.750	0.020	0.230	0.608	0.150	0.242	0.962	0.015	0.023
61	0.763	0.030	0.207	0.612	0.130	0.258	0.911	0.049	0.040
62	0.531	0.195	0.274	0.388	0.301	0.311	0.005	0.506	0.489
63	0.601	0.128	0.271	0.432	0.265	0.303	0.002	0.578	0.420
64	0.696	0.071	0.233	0.502	0.229	0.269	0.000	0.614	0.386
65	0.703	0.054	0.243	0.527	0.213	0.260	0.000	0.670	0.330
66	0.692	0.049	0.259	0.549	0.214	0.237	0.000	0.680	0.320
67	0.681	0.053	0.266	0.560	0.208	0.232	0.000	0.672	0.328
68	0.693	0.051	0.256	0.557	0.205	0.238	0.000	0.675	0.325
69	0.711	0.051	0.238	0.583	0.183	0.234	0.000	0.692	0.308
70	0.700	0.204	0.096	0.643	0.264	0.093	0.016	0.672	0.312
71	0.688	0.045	0.267	0.547	0.209	0.244	0.000	0.679	0.321
72	0.828	0.019	0.153	0.533	0.328	0.139	0.001	0.722	0.277
73	0.926	0.013	0.061	0.488	0.464	0.048	0.000	0.910	0.090
74	0.975	0.006	0.019	0.022	0.978	0.000	0.000	0.988	0.012
75	0.979	0.004	0.017	0.014	0.986	0.000	0.000	0.988	0.012
76	0.598	0.002	0.400	0.404	0.053	0.543	0.056	0.148	0.796
77	0.668	0.035	0.297	0.551	0.181	0.268	0.000	0.653	0.347
78	0.653	0.037	0.310	0.537	0.183	0.280	0.000	0.639	0.361
79	0.659	0.026	0.315	0.501	0.181	0.318	0.000	0.622	0.378
80	0.204	0.002	0.794	0.096	0.072	0.832	0.000	0.223	0.777
81	0.073	0.001	0.926	0.031	0.044	0.925	0.000	0.092	0.908
82	0.213	0.002	0.785	0.104	0.063	0.833	0.000	0.221	0.779
83	0.061	0.001	0.938	0.017	0.022	0.961	0.000	0.077	0.923
84	0.769	0.021	0.210	0.740	0.063	0.197	0.000	0.791	0.209
85	0.712	0.046	0.242	0.558	0.208	0.234	0.000	0.676	0.324
86	0.788	0.008	0.204	0.606	0.283	0.111	0.000	0.730	0.270
87	0.687	0.000	0.313	0.084	0.862	0.054	0.000	0.704	0.296
88	0.986	0.000	0.014	0.004	0.996	0.000	0.000	0.984	0.016
89	0.740	0.014	0.246	0.588	0.153	0.259	0.000	0.760	0.240
90	0.740	0.015	0.245	0.592	0.161	0.247	0.000	0.757	0.243
91	0.744	0.017	0.239	0.595	0.117	0.288	0.000	0.739	0.261
92	0.625	0.007	0.368	0.474	0.058	0.468	0.000	0.593	0.407
93	0.988	0.003	0.009	0.982	0.003	0.015	0.030	0.792	0.178
94	0.993	0.002	0.005	0.992	0.001	0.007	0.884	0.077	0.039
95	1.000	0.000	0.000	1.000	0.000	0.000	1.000	0.000	0.000
96	0.721	0.010	0.269	0.570	0.117	0.313	0.000	0.741	0.259
97	0.871	0.007	0.122	0.816	0.041	0.143	0.000	0.880	0.120

State of character:

0 = absence (white)

1 = presence (blue)

2= no petals (black)

Ancestral state reconstructions without zonation

Invagination on abaxial side

	0	1	2
50	0.288	0.453	0.259
51	0.441	0.352	0.207
52	0.534	0.242	0.224
53	0.541	0.188	0.271
54	0.559	0.183	0.258
55	0.537	0.182	0.281
56	0.580	0.164	0.256
57	0.595	0.149	0.256
58	0.558	0.165	0.277
59	0.553	0.206	0.241
60	0.572	0.156	0.272
61	0.599	0.141	0.260
62	0.398	0.444	0.158
63	0.379	0.459	0.162
64	0.351	0.522	0.127
65	0.349	0.521	0.130
66	0.349	0.534	0.117
67	0.343	0.548	0.109
68	0.332	0.566	0.102
69	0.228	0.678	0.094
70	0.047	0.937	0.016
71	0.379	0.500	0.121
72	0.397	0.456	0.147
73	0.334	0.599	0.067
74	0.003	0.996	0.001
75	0.002	0.998	0.000
76	0.600	0.007	0.393
77	0.492	0.351	0.157
78	0.517	0.305	0.178
79	0.541	0.229	0.230
80	0.633	0.011	0.356
81	0.557	0.004	0.439
82	0.620	0.010	0.370
83	0.519	0.004	0.477
84	0.630	0.094	0.276
85	0.393	0.469	0.138
86	0.622	0.082	0.296
87	0.664	0.001	0.335
88	0.951	0.000	0.049
89	0.555	0.241	0.204
90	0.538	0.243	0.219
91	0.523	0.281	0.196
92	0.666	0.029	0.305
93	0.034	0.964	0.002
94	0.034	0.964	0.002
95	0.000	1.000	0.000
96	0.598	0.139	0.263
97	0.669	0.045	0.286

Outgrowth on adaxial side

	0	1	2
50	0.343	0.334	0.323
51	0.465	0.274	0.261
52	0.493	0.256	0.251
53	0.514	0.234	0.252
54	0.505	0.261	0.234
55	0.497	0.241	0.262
56	0.478	0.252	0.270
57	0.520	0.231	0.249
58	0.480	0.254	0.266
59	0.520	0.232	0.248
60	0.499	0.235	0.266
61	0.515	0.218	0.267
62	0.451	0.256	0.293
63	0.501	0.229	0.270
64	0.506	0.233	0.261
65	0.505	0.262	0.233
66	0.517	0.233	0.250
67	0.523	0.237	0.240
68	0.515	0.219	0.266
69	0.553	0.196	0.251
70	0.501	0.237	0.262
71	0.517	0.238	0.245
72	0.505	0.244	0.251
73	0.563	0.216	0.221
74	0.824	0.067	0.109
75	0.833	0.079	0.088
76	0.500	0.228	0.272
77	0.500	0.246	0.254
78	0.514	0.233	0.253
79	0.522	0.214	0.264
80	0.435	0.282	0.283
81	0.359	0.345	0.296
82	0.449	0.291	0.260
83	0.326	0.331	0.343
84	0.527	0.229	0.244
85	0.493	0.229	0.278
86	0.539	0.238	0.223
87	0.582	0.215	0.203
88	0.934	0.026	0.040
89	0.474	0.266	0.260
90	0.476	0.256	0.268
91	0.499	0.247	0.254
92	0.347	0.324	0.329
93	0.717	0.133	0.150
94	0.712	0.139	0.149
95	0.988	0.006	0.006
96	0.480	0.238	0.282
97	0.463	0.251	0.286

Claw

	0	1	2
50	0.076	0.400	0.524
51	0.627	0.154	0.219
52	0.926	0.031	0.043
53	0.949	0.027	0.024
54	0.949	0.028	0.023
55	0.948	0.029	0.023
56	0.958	0.023	0.019
57	0.964	0.019	0.017
58	0.943	0.030	0.027
59	0.950	0.021	0.029
60	0.976	0.011	0.013
61	0.910	0.054	0.036
62	0.010	0.516	0.474
63	0.002	0.587	0.411
64	0.000	0.663	0.337
65	0.000	0.686	0.314
66	0.000	0.683	0.317
67	0.000	0.696	0.304
68	0.000	0.689	0.311
69	0.000	0.702	0.298
70	0.012	0.680	0.308
71	0.000	0.679	0.321
72	0.000	0.729	0.271
73	0.000	0.931	0.069
74	0.000	0.991	0.009
75	0.000	0.992	0.008
76	0.043	0.165	0.792
77	0.000	0.664	0.336
78	0.000	0.662	0.338
79	0.000	0.632	0.368
80	0.000	0.207	0.793
81	0.000	0.095	0.905
82	0.000	0.217	0.783
83	0.000	0.067	0.933
84	0.000	0.784	0.216
85	0.000	0.692	0.308
86	0.000	0.770	0.230
87	0.000	0.702	0.298
88	0.000	0.984	0.016
89	0.000	0.764	0.236
90	0.000	0.768	0.232
91	0.000	0.725	0.275
92	0.000	0.597	0.403
93	0.042	0.792	0.166
94	0.889	0.089	0.022
95	0.999	0.001	0.000
96	0.000	0.745	0.255
97	0.000	0.858	0.142
