# Supplementary material for: A flat petal as ancestral state for Ranunculaceae
Source: Front Plant Sci. 2022 Sep 21;13:961906. doi: 10.3389/fpls.2022.961906 (PMC9532948; doi:10.3389/fpls.2022.961906)
Supplement: Supplementary file 11 [file Data_Sheet_11.pdf]

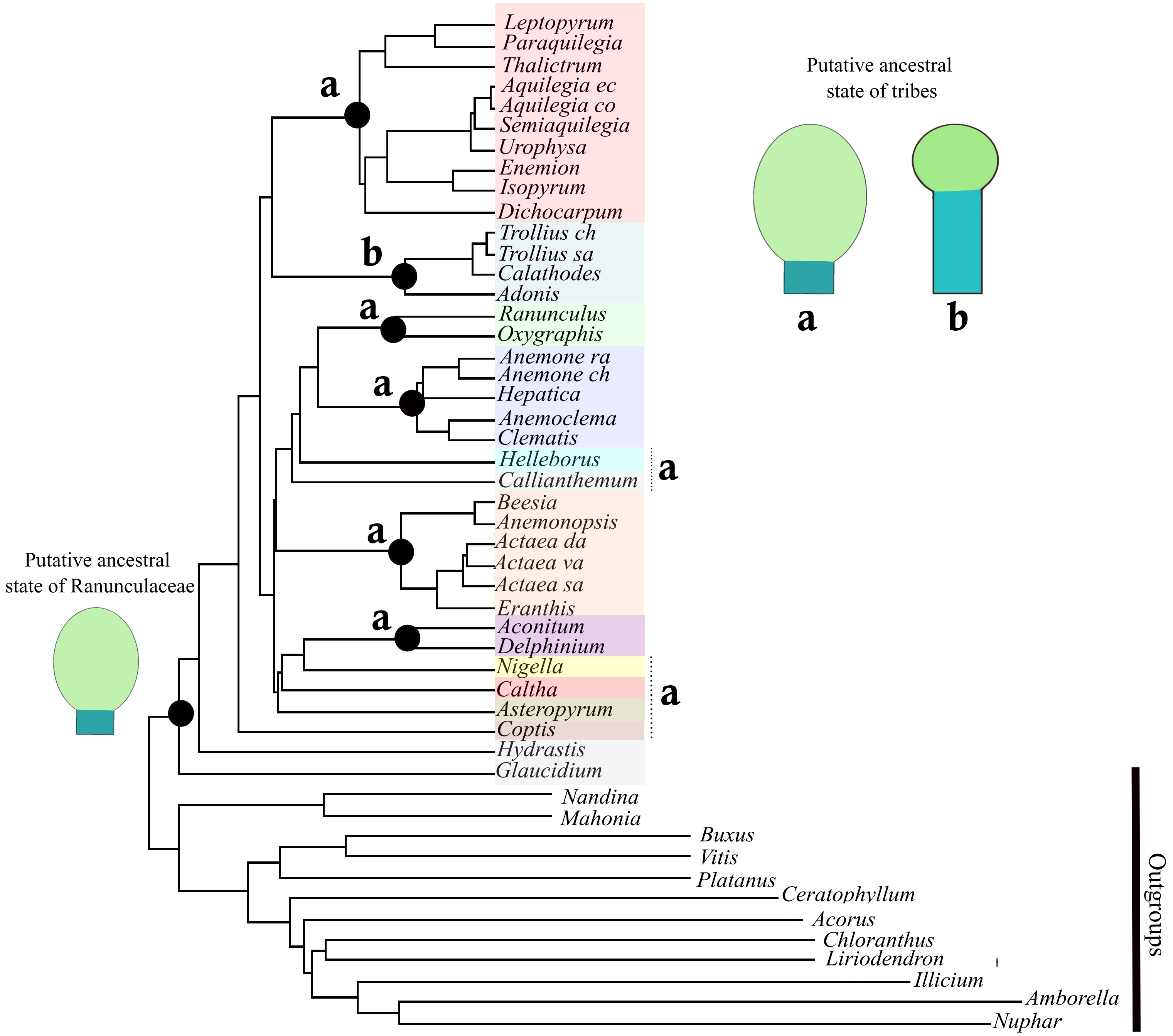

Supplementary material 11: Putative ancestral petal shape (a and b) for the family as a whole and for the different tribes according to the results of ancestral state reconstructions of each character for each zone of the petal. The colour code for tribes is the same as in figure 1.
